# 16S rRNA Gene Amplicon Based Metagenomic Signatures of Rhizobiome Community in Rice Field During Various Growth Stages

**DOI:** 10.3389/fmicb.2019.02103

**Published:** 2019-09-20

**Authors:** Madangchanok Imchen, Ranjith Kumavath, Aline B. M. Vaz, Aristóteles Góes-Neto, Debmalya Barh, Preetam Ghosh, Natalia Kozyrovska, Olga Podolich, Vasco Azevedo

**Affiliations:** ^1^Department of Genomic Sciences, School of Biological Sciences, Central University of Kerala, Kasaragod, India; ^2^Molecular and Computational Biology of Fungi Laboratory, Department of Microbiology, Institute of Biological Sciences, Federal University of Minas Gerais, Belo Horizonte, Brazil; ^3^Centre for Genomics and Applied Gene Technology, Institute of Integrative Omics and Applied Biotechnology (IIOAB), Purba Medinipur, India; ^4^Department of Computer Sciences, Virginia Commonwealth University, Richmond, VA, United States; ^5^Institute of Molecular Biology and Genetics, National Academy of Sciences, Kyiv, Ukraine; ^6^Laboratório de Genética Celular e Molecular, Departamento de Biologia Geral, Instituto de Ciências Biológicas, Universidade Federal de Minas Gerais, Belo Horizonte, Brazil

**Keywords:** rhizobiome, *Oryza sativa*, environmental DNA, 16S rRNA gene amplicon metagenomics, next generation sequencing

## Abstract

Rice is a major staple food across the globe. Its growth and productivity is highly dependent on the rhizobiome where crosstalk takes place between plant and the microbial community. Such interactions lead to selective enrichment of plant beneficial microbes which ultimately defines the crop health and productivity. In this study, rhizobiome modulation is documented throughout the development of rice plant. Based on 16S rRNA gene affiliation at genus level, abundance, and diversity of plant growth promoting bacteria increased during the growth stages. The observed α diversity and rhizobiome complexity increased significantly (*p* < 0.05) during plantation. PCoA indicates that different geographical locations shared similar rhizobiome diversity but exerted differential enrichment (*p* < 0.001). Diversity of enriched genera represented a sigmoid curve and subsequently declined after harvest. A major proportion of dominant enriched genera (*p* < 0.05, abundance > 0.1%), based on 16S rRNA gene, were plant growth promoting bacteria that produces siderophore, indole-3-acetic acid, aminocyclopropane-1-carboxylic acid, and antimicrobials. Hydrogenotrophic methanogens dominated throughout cultivation. Type I methanotrophs (*n* = 12) had higher diversity than type II methanotrophs (*n* = 6). However, the later had significantly higher abundance (*p* = 0.003). Strong enrichment pattern was also observed in type I methanotrophs being enriched during water logged stages. Ammonia oxidizing Archaea were several folds more abundant than ammonia oxidizing bacteria. K-strategists *Nitrosospira* and *Nitrospira* dominated ammonia and nitrite oxidizing bacteria, respectively. The study clarifies the modulation of rhizobiome according to the rice developmental stages, thereby opening up the possibilities of bio-fertilizer treatment based on each cultivation stages.

## Introduction

Soil comprises mineral particles of different shapes and sizes with distinct chemical characteristics and organic compounds in various stages of decomposition. Molecular studies suggest that soil harbor the highest biodiversity of organisms on Earth, approximately 1,000 Gbp of microbial genome sequences per gram of soil (Vogel et al., 2009). These microorganisms play important roles in maintenance of soil fertility, nutrient cycling, and carbon sequestration. The rhizosphere is the soil around living roots influenced by root activity (Hartmann et al., 2008); the chemical composition of the exudates, border cells and mucilage released by plant roots are important to signal and also establish the microbial community in this environment (Mendes et al., 2013). The rhizobiome can influence the plant directly and/or indirectly (Schnitzer et al., 2011), as seen by the PGPB (plant growth promoting bacteria), which acts synergistically on plant growth promotion and disease suppression (Liu et al., 2018). The microbial diversity present in rhizosphere of native and cultivated crops can be different due to the species-specific effects (Philippot et al., 2013). For example, the bulk microbiome is more complex than rhizobiome of *Dendranthema grandiflora Tzvelev* (chrysanthemum) (Duineveld et al., 2001), sagebrush (Gonzalez-Franco et al., 2009), and oak (Uroz et al., 2010). On the other hand, the opposite trend was observed for other plants, such as wheat (Donn et al., 2015), wild oat (Shi et al., 2016), and switch grass (He et al., 2017). Additionally, the root exudate composition changes during plant growth and influence microbial community assembly in the rhizosphere, as shown in *Arabidopsis* (Chaparro et al., 2014), soybean (Sugiyama et al., 2014), grapevine (Novello et al., 2017), and maize (Walters et al., 2018) rhizosphere. The microbial community assembly in rhizosphere is also determined by the abiotic and biotic factors influencing both natural and agricultural ecosystems (Philippot et al., 2013). Studies performed in greenhouse showed differences in the rice rhizobiome cultivated in soils with different fertilizer (Innes et al., 2004) and in rice-wheat rotation cultivation in the field (Wang et al., 2016).

Rice is the major stable food for more than half of the world’s population, and more than half of the supply comes from India and China (Muthayya et al., 2014). Due to the high consumption of this crop, rice paddies occupy around 11% of the arable land in the world. However, rice cultivation is responsible for 20% of total agricultural CH_4_ emission (Van Groenigen et al., 2011). High greenhouse-gas (GHG) emission occurs by the growth of methanogenic archaea present in the vicinity of rice roots (Neue, 1993). On the other hand, the increase of CO_2_ and temperature will elevate the CH_4_ emission, indicating a positive feedback loop. This suggests that higher photosynthetic rate results in an increase of substrates availability for the methanogenesis (Tokida et al., 2010). The release of CH_4_ (methane) into the atmosphere is further aided by the rice vascular system, which serves as a passage for a part of CH_4_ from soil to the atmosphere, bypassing the methanotrophic community (Hernández et al., 2015). The anaerobic conditions in paddy rice also preclude the nitrification, decreasing the NO^3–^ production, and promoting the complete denitrification of NO^3–^ to N_2_, resulting in low N_2_O emission (Verhoeven et al., 2018).

Although scientific understanding about the diversity and composition of soil microbiome can directly and/or indirectly influence a wide range of ecosystem processes, few efforts have been directed toward understanding the microbial biogeography (Fierer and Jackson, 2006). To address this question, we have undertaken a spatial and temporal characterization of rhizobiome and bulk soil from rice paddy using targeted amplicon-based (16S rRNA gene) metagenomic approach, and we posit the following predictions: the microbial community (i) is significantly different in bulk and rhizosphere soils, (ii) changes across the rice cultivation stages, and (iii) is distinct according to the geographical scale analyzed.

## Materials and Methods

### Sampling and Environmental DNA Isolation

The rhizosphere and bulk soil samples were collected during the month of December 2016 to April 2017 in seven different India states (Table 1 and Supplementary Figure S1). All of them are situated in rural areas away from any industrial set up in the vicinity. All the sites practiced rice monoculture for more than 10 years and during off-season are kept as fallow land, and cow manure was used after plowing. Ammonium, phosphate, and sulfate (Potash, FACTAMFOS) were used during seedling stage. The rice rhizosphere soil was sampled in triplicates by pulling out the plant to collect the soil loosely attached to the roots from three different geographical sites in all growth stages: vegetative, reproductive and ripening. Moreover, bulk soil was also collected in pre-plow, post-plow, and after harvest stages. To investigate the relationships among the bacterial community similarity and geographical distance, additional samples were taken from vegetative, and post-plow stages in paddy fields in different sites in India (Table 1). All the samples were collected from upper 5 – 15 cm in 5 replicate subsamples, which were maintained in individualized sterile plastic bags. The environmental DNA was extracted using HiPurA^TM^ Soil DNA purification kit (HiMedia) following the manufacturer’s protocol. In order to increase the DNA yield, the bead-beating step was extended to 15 min, and incubation during elution step was extended to 30 min at 37°C. Soil pH was measured, and ammonia and nitrite were analyzed using 2M potassium chloride extract as described elsewhere (Klotz and Stein, 2011).

**TABLE 1:** Description of study sites.

**Geographic coordinates**	**Sampling stage (samples code)**
Vadakara, Kerala (*11*°*33′42.4″N 75*°*39′28.3″E*)	Pre-plow (kvp1) Post-plow (kvp2) Vegetative (kvv) Reproductive (kvr1) Ripening (kvr2) Post-harvest (kvph)
Chemmattamvayal, Kerala (*12*°*18′48.0″N 75*°*06′30.2″E*)	Pre-plow (kcp1) Post-plow (kcp2) Vegetative (kcv) Reproductive (kcr1) Ripening (kcr2) Post-harvest (kcph)
Kheezmala, Kerala (*12*°*16′50.1″N 75*°*12′42.7″E*)	Pre-plow (kkp1) Post-plow (kkp2) Vegetative (kkv) Reproductive (kkr1) Ripening (kkr2) Post-harvest (kkph)
Dimapur, Nagaland (*25*°*54′52.7″N 93*°*47′18.6″E*)	Vegetative (nl)
Rasipuran, Tamil Nadu (*11*°*26′40.5″N 78*°*10′26.6″E*)	Vegetative (tn)
Ukhurl, Manipur (*25*°*05′45.6″N 94*°*21′19.3″E*)	Vegetative (mn)
Burari, Delhi (*28*°*46′26.9″N 77*°*12′11.9″E*)	Post-plow (dl)
Panvel, Maharashtra (*19*°*01′01.4″N 73*°*07′32.1″E*)	Post-plow (mh)
Mysore, Karnataka (*12*°*30′39.4″N 76*°*54′05.7″E*)	Post-plow (my)

### 16S rRNA Gene Amplification and Next Generation Sequencing

The prokaryotic hypervariable V3–V4 region from 16S rRNA gene was amplified using the primers set Pro341F (5′ – CCTACGGGNBGCASCAG – 3′) and Pro805R (5′- GACTACNVGGGTATCTAATCC- 3′) (Takahashi et al., 2014). Equimolar quantities of PCR amplicon in replicates were pooled and sequenced using Illumina MiSeq platform at Eurofins Scientific (Bangalore, India). The raw fastQ files were uploaded to the metagenome rapid annotation using subsystem technology (MG-RAST) server (Meyer et al., 2008) and annotated using default parameters. Briefly, the pipeline removes low quality sequences having phred scores below 15 (Cox et al., 2010). Artificial duplicate reads were removed using DRISEE (duplicate read inferred sequencing error estimation) (Gomez-Alvarez et al., 2009). The sequences were annotated using the RDP (Ribosomal Database Project) database having a minimum cutoff identity of 60% and *e*-value of 5. All samples are publicly available at MG-RAST under the IDs provided in Supplementary Table S1.

### Statistical Methods

The alpha diversity was estimated by observed richness, Chao1, and Shannon diversity. The diversity was estimated using Shannon (*H*′) index [*H*′ = −Σ*ni/n ln* (*ni/n*), where *ni* is the number of individuals in the taxon *i* and *n* is the total number of individuals], which is influenced by both species richness and evenness. Difference in alpha diversity in various growth stages was calculated using Kruskal Wallis. Rarefaction curves were plotted to estimate whether the sequencing depth is enough to wholly capture the microbial diversity. Statistical difference in bacterial composition based on plantation, cultivation stages and geographical locations was calculated using PERMANOVA (permutational multivariate analysis of variance using distance matrices) and Bray Curtis distance. The *p* values were based on 999 permutations, and the *adonis* function of vegan package was used. Taxonomical network was constructed in R (R Core Team, 2014) with a jaccard maximum distance of 0.05 and 0.5 through *make_network* function from phyloseq v1.16.2 package. Network visualization was performed in Gephi-0.9.2 (Bastian et al., 2009). The samples were clustered in two groups, cropped (vegetative, reproductive, and ripening stages) and uncropped (pre-plow, post-plow, and post-harvest steps).

## Results

Soil analysis for each growth stages showed a consistent pH of 4 – 6. Similarly, growth stages also had a little effect on nitrite concentration ranging from 0.8 to 1.2 μM having higher fluctuation between samples during the growth stages (Supplementary Figure S2). However, ammonia concentration was quiet distinguishable between uncropped (15 – 30 μM) and cropped (25 – 45 μM) stages showing almost 2× fold increase once the plant reached reproductive stage. High-throughput sequencing of the 16S rRNA gene amplicons generated more than 6.53 Gb, representing a total of 7.1 million reads. Over 207.825.194 bp of the total reads passed quality control (Supplementary Table S1). The high-quality reads were clustered using >97% sequence identity into 3856 microbial OTUs. The rarefaction curves reached a stable asymptote for all groups (Supplementary Figure S3). A significant portion of the microbiome fell under uncultured/unclassified taxa (Supplementary Table S2).

### Soil Microbial Community Differs in Rhizosphere Across Rice Cultivation

**FIGURE 1:**
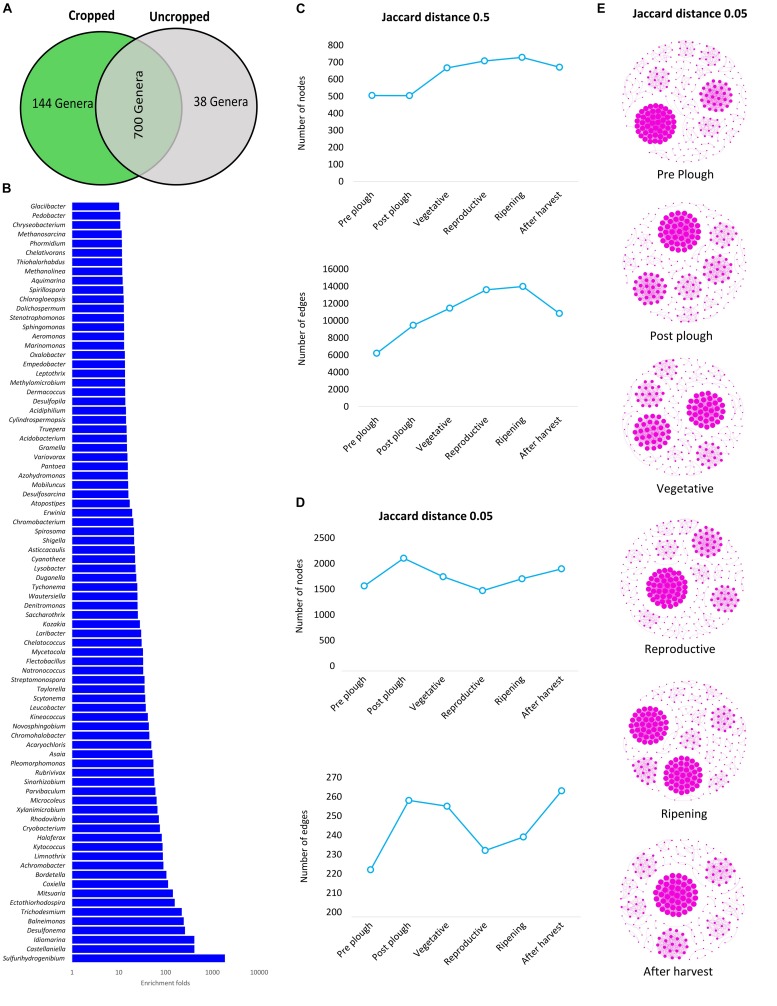
**(A)** Venn diagram of cropped and uncropped microbiome shows a high similarity of abundance. **(B)** Genera with more than 10 folds enrichment in rhizobiome. **(C,D)** Number of nodes and edges for 0.5 and 0.05-jaccard distance, respectively. **(E)** 0.05 jaccard distance network complexity map.

**FIGURE 2:**
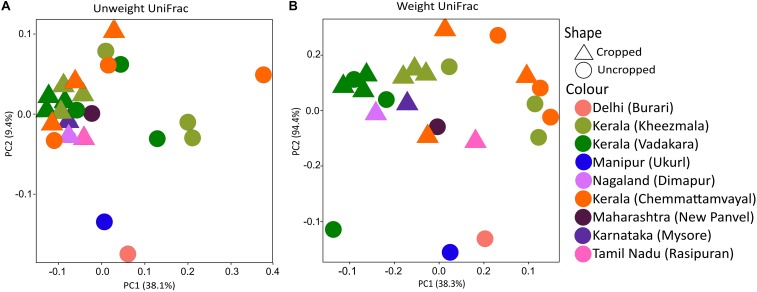
PCoA rhizobiome analysis of different cultivation stages and location using **(A)** unweight unifrac shows clustering of cropped samples irrespective of location and cultivation stage. In contrast, **(B)** weight unifrac PCoA shows culturing majorly based on location implying that the diversity is relatively similar, but not abundance, among the samples during cropped stages.

Twenty-eight phyla were obtained from all groups (Supplementary Figure S4) and the most abundant were Proteobacteria (25.69 ± 13.92%), Firmicutes (20.82 ± 8.69%), Actinobacteria (16.68 ± 5.93%), and Acidobacteria (13.28 ± 10.47%). The rhizosphere had a higher number of genera (*n* = 844) than the bulk soil (*n* = 735), and both of them shared 79.36% of these genera (*n* = 700) (Figure 1A). Twenty-three genera showed more than 1% of occurrence from any sample (Supplementary Figure S5). The most abundant genus from rhizosphere group was a *Candidatus Koribacter* (7.5–18.6%) while the most frequently found genus in bulk soil samples was *Ktedonbacter* (13.4%). Measures of within sample diversity (alpha diversity) showed a gradient from bulk soil to rhizosphere samples; however, the difference in alpha-diversity could not be considered statistically significant (Table 2). PERMANOVA analysis showed that the community composition of the bulk soil was statistically different (Table 3) and higher complex network structure than rhizosphere (Figures 1C–E). Pairwise comparison of community dissimilarity variances showed that significant differences between post-plow to ripening and post-plow to post-harvest groups (Table 4). The community structure was also statistically different considering geographic location (Table 3), which was corroborated by weighted unifrac PCoA (principal coordinates analysis) (Figure 2B). On the other hand, unweighted PCoA analysis showed the separation of rhizosphere and bulk soil samples irrespective of geography, indicating similarities in the microbial composition instead of abundance (Figure 2A).

**TABLE 2:** Pairwise comparisons of alpha diversity between each stage.

	**Pre-plow**	**Post-plow**	**Vegetative**	**Reproductive**	**Ripening**	**Uncropped**
Post-plow	1.00					
Vegetative	0.62	0.90				
Reproductive	0.90	0.33	1.00			
Ripening	0.90	0.33	0.90	1.00		
Post harvest	1.00	1.00	1.00	1.00	1.00	
Cropped						0.005

*Hypothesis testing was carried out using Wilcoxon rank sum tests and corrected for multiple testing using the Benjamini-Hochberg method.*

**TABLE 3:** Permutational multivariate analysis of variance using distance matrices (PERMANOVA) of bulk and rhizosphere microbiome compared to cropping, growth stages and location.

**Group comparisons**	**Bray**		**Manhattan**		**Canberra**		**Jaccard**	
				
	***R*^2^**	***P* value**	***R*^2^**	***P* value**	***R*^2^**	***P* value**	***R*^2^**	***P* value**
Cropped vs. Uncropped	0.102	0.0002	0.102	0.0005	0.074	0.0004	0.083	0.0015
Cultivation stages	0.162	0.0193	0.162	0.0173	0.181	0.0174	0.162	0.0304
Geographical location	0.505	0.0003	0.505	0.0003	0.384	0.0006	0.478	0.0001

**TABLE 4:** Significant differences in microbial community among the groups using ANOSIM.

	**Pre-plow**	**Post-plow**	**Vegetative**	**Reproductive**	**Ripening**	**Post-harvest**
Pre-plow		0.22	0.08	0.26	0.33	–0.22
Post-plow	0.14		0.02	0.27	0.40	0.5
Vegetative	0.32	0.36		–0.22	–0.07	0.06
Reproductive	0.20	0.14	0.91		0.15	–0.04
Ripening	0.33	0.046	0.57	0.5		0.19
Post-harvest	1	0.027	0.36	0.6	0.1	

*The superior part of the table corresponds to the *R*-value and the inferior part to the *P*-value. The *P*-values indicated the significant differences at the levels of *p* < 0.05. Cropped vs. Uncropped = *R*-value: 0.091, *P* = 0.057.*

### Growth Stage-Dependent Variations in Microbial Community

**FIGURE 3:**
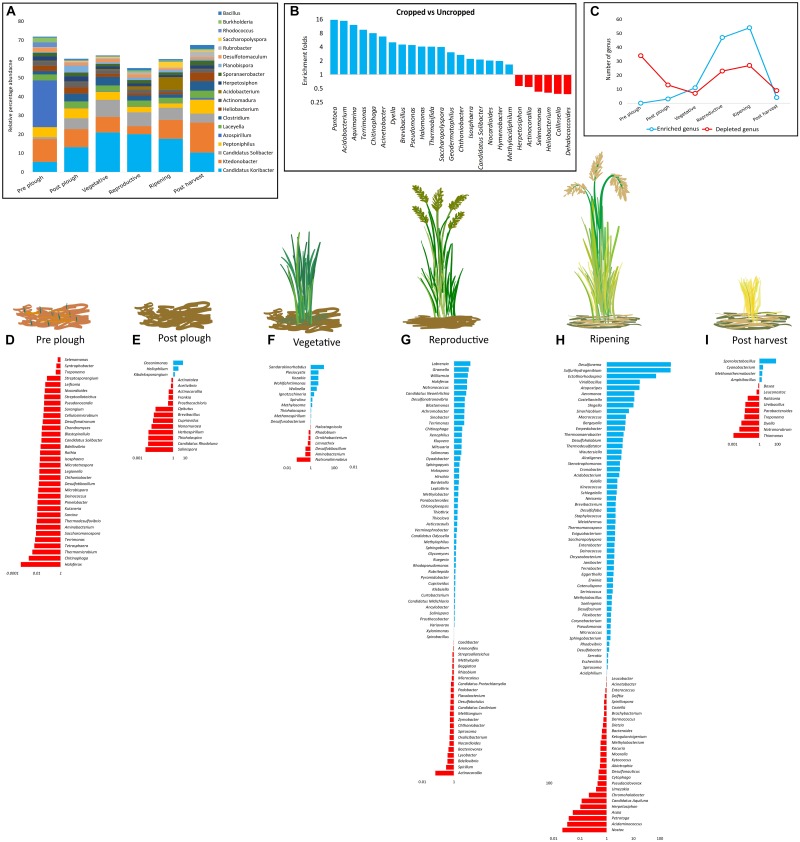
Growth stage-dependent selective enrichment **(A)** at genus level of the top 10 most abundant genera from each cultivation stage. **(B)** Enrichment and depletion on a larger time frame (cropped vs. uncropped). **(C–I)** Growth stage-dependent genus enrichment and depletion (*p* < 0.05). Blue color denotes enrichment, red color denotes depletion.

All genera (*n* = 34) that were significantly (*p* < 0.05) altered in pre-plow stage were suppressed (Figure 3D). Similarly, several genera were suppressed in post-plow stage, except for *Kibdelosporangium*, *Heliophilum*, and *Oceanimonas* (Figure 3E). During vegetative stage, number of enriched genera increased further (*n* = 11) while the percentage of the depleted genera reduced (39%) (Figure 3F). The richness and abundance of enriched genera increased while the percentage of the down-regulated genera reduced with plant growth (Figures 3C–I). Interestingly, majority of the genera that experienced significant changes during plant growth were up-regulated while those genera with significant changes before plantation or after harvest were depleted. In order to catalog rhizobiome growth stage-dependent enriched genera in each location, all genera having above 1% abundance in any cultivation stage were screened to obtain the dominant genera and a heatmap was constructed (Supplementary Figure S6). In line to the weight PCoA, rhizobiome enrichment had more difference than similarities among the location. Several genera such as *Ca*. Koribacter, *Ca*. Solibacter, *Clostridium, Pseudonocardia, Geoalkalibacter, Saccharopolyspora*, and *Acidobacterium* were enriched commonly during the plantation in all locations. However, among those common genera, there was no consistent enrichment patterns with respect to growth stage between the locations. For instance, *Ca*. Koribacter was enriched during vegetative and ripening stage at site KK, while the same genus was enriched during reproductive stage and post-harvest stage for site KC and KV, respectively. Similarly, *Ca.* Solibacter was enriched during all the cultivation stages at site KK and KV while it was enriched majorly at reproductive stage in site KC. The highly abundant genus, *Ktedonobacter*, was depleted gradually as the cultivation progressed at site KK and KC, which was re-enriched only after post-harvest. However, it was enriched at site KV during post-harvest, as well as vegetative stage.

### Methane Metabolism and Nitrification

**FIGURE 4:**
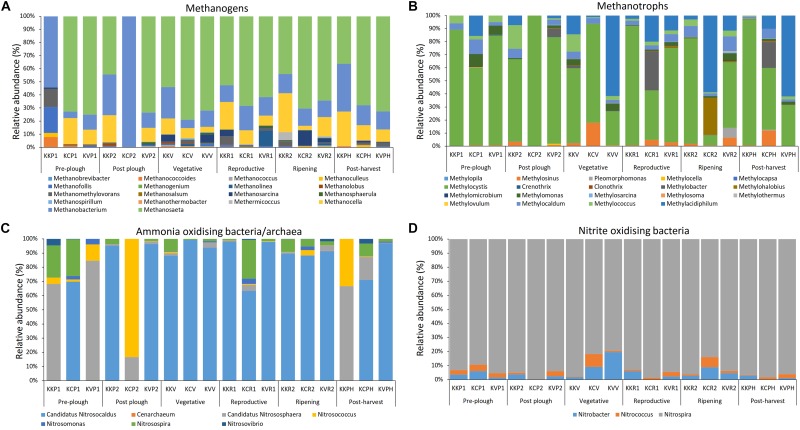
Rhizobiome composition and diversity of **(A)** methanogens, **(B)** methanotrophs, **(C)** ammonia oxidizers, and **(D)** nitrite oxidizers in a stage wise manner shows differential enrichment within respective niche throughout the cultivation.

Eighteen methanogen genera were found in all samples, which accounted for 0.96 – 1.35% of overall abundance in each stage. The abundance rose sharply post-plow probably due to the application of organic cow manure, and declined with plant age until post-harvest. Dominant genera were *Methanosaeta*, followed by *Methanobacterium* and *Methanocella* (Figure 4A and Supplementary Figure S7). Archaeal genera belonging to type I and type II methanotrophs were present throughout the cultivation. Abundance of *Methanosarcina* was constant until vegetative period, decreased at reproductive (*p* < 0.046), and increased at ripening stage (*p* < 0.046). *Methermicoccus* abundance increased at ripening stage and decreased after harvest. Predominant methanotroph genera belonging to first group were *Methylococcus* and *Methylocaldum*, while *Methylocystis* and *Methylosinus* were predominant in the second group (Figure 4B and Supplementary Figure S8). There was a significant (*p* = 0.003) difference between type I and type II methanotroph abundance. Methanotrophs belonging to the Verrucomicrobial phylum, previously considered as extremophiles, were also enriched during the cropped and post-harvest stages. Ammonia concentration increased significantly at reproductive stage, including ripening and post-harvest (26 – 48 μM) in contract to the initial growth stages (19 – 28 μM). However, no significant differences were observed between bulk soil and rhizosphere groups based on either ammonia or on nitrite concentration (Supplementary Figure S9). AOA (ammonia-oxidizing Archaea) dominated over AOB (ammonia-oxidizing bacteria) throughout the cultivation ranging from 3 to 30 folds (*p* = 0.003) (Figure 4C). Genera *Candidatus* Nitrosocaldus and *Nitrosospira* were the dominant members of AOA and AOB, respectively. Within the nitrite oxidizers, *Nitrospira* was the most dominant followed by *Nitrobacter* and *Nitrococcus* (Figure 4D). Type I methanotrophs, nitrite oxidizers, and AOA showed a similar pattern during the ripening stage having reduced abundance while AOB increased.

## Discussion

### Alpha Diversity Increases Gradually in Cropped Rhizosphere During Plantation

**FIGURE 5:**
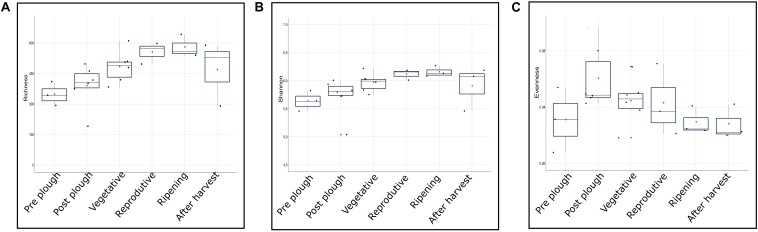
Growth stage-dependent **(A)** richness, **(B)** Shannon, and **(C)** evenness represented in box plot.

Higher alpha diversity have been reported in rhizosphere, rather than bulk soil, of rice (Edwards et al., 2015), maize (Yang et al., 2017), and cotton (Qiao et al., 2017). In contrast, studies have also highlighted higher alpha diversity in bulk soil, rather than rhizosphere, in maize (Peiffer et al., 2013), soy plant (Wang P. et al., 2017), and annual grass *Avena fatua* (Shi et al., 2015). Cultivation practice such as rotation crop system of alfalfa-rice, wheat-rice (Lopes et al., 2014) and continuous monocropping of black pepper (Xiong et al., 2015) exhibited higher alpha diversity in uncropped soil. Vegetative stage of rice rhizosphere during flooded condition had higher alpha diversity compared to reproductive (matured) stage (Pittol et al., 2018). Such contrasting literature shows a missing gap between rhizosphere and bulk soil alpha diversity. In this study, gradual increase of alpha diversity was observed with plant age irrespective of location or genotype (Figures 5A–C). During the reproductive and ripening stage, the alpha diversity had the lowest standard deviation indicating a stable diversity. Alpha diversity between any two consecutive stages didn’t show significant difference (*P* > 0.05) (Table 2). However, comparison of alpha diversity between rhizosphere and uncropped soil showed statistically significant difference in observed alpha diversity (*P* < 0.05). This indicates that the enrichment in rhizosphere alpha diversity is a gradual process with no significant changes from one cropping stage to another, however, the changes are prominent when viewed at a larger timeframe i.e., cropped vs. uncropped.

### Rhizosphere Microbial Community Is Diverse and Complex Yet Specialized

Denaturing gradient gel electrophoresis (DGGE) studies on *Dendranthema grandiflora* Tzvelev (chrysanthemum) have indicated rhizosphere as a subset of bulk soil (Duineveld et al., 2001). These observations are further backed up by NGS based metagenomics studies on soybean (Mendes et al., 2014) having lesser network complexity in the rhizosphere relative to bulk soil. However, rhizosphere of wheat and wild oat had microbial diversity increased with plant age (Donn et al., 2015) and markedly more complex than the surrounding bulk soil although the diversity was decreased (Shi et al., 2016). Cultivation of switch-grass also showed an increased microbial network structure in rhizosphere (He et al., 2017). In this study, community co-occurrence network was highly dependent on the threshold maximum distance between the nodes. The number of nodes (vertices), as well as the edges (links) of the co-occurrence network increased with plant growth with a strong correlation (*r* = 0.9, *p* < 0.03) between nodes and edges when the maximum jaccard distance between the nodes were set to 0.5. Although the number of nodes remained similar pre and post-plow (Figures 1C–E), the edges increased ∼1.5 folds post-plow indicating the increased association among the genera. During the cultivation period, the complexity (nodes and edges) increased in every succeeding stage, which continued until after-harvesting stage corroborating to the high alpha diversity. High correlation was found between the number of nodes and edges against alpha diversity (*r* = 0.58 – 0.95; *p* = 0.003 – 0.066). In contrast, network map having maximum distance of 0.05 had number of nodes as well as edges decreased significantly during the cropped stages (Figures 1D,E and Supplementary Figure S10). The contrasting observation in network map indicates that the microbial network in rhizosphere constitutes a higher number of complex relationships compared to the bulk soil; however, they share a lower number of strong linked relationships. This decline of strong linked relationships could be due to selective enrichment of beneficial microbes exerted by the root exudates which leads to partial depletion of non-beneficial microbes. This also indicates that the cropped and uncropped soil samples shared a common pool and the selective enrichment is highly regulated by the root exudates. In support of this, Venn diagram of the cropped and uncropped samples indicated that the number of genera unique to cropped stages increased; however, they constituted merely 0.626% of the overall abundance (Figure 1A). Meanwhile the common genera (*n* = 700) between cropped and uncropped stages accounted for over 99% of the abundance. A closer look at each genus showed high enrichment of selected genus (Figure 1B). This indicates that the rhizosphere microbiome, in addition to high diversity and complexity, is highly selective in rhizobiome recruitment.

### Geography Influences Community Abundance

Physiological factors such as growth stages have shown relatively weak influence over determination of the rice rhizobiome and highlighted the importance of geography in shaping the rice rhizosphere microbial community (Edwards et al., 2015). However, in such studies, the geographical location distances were relatively small and hence a larger variation is required as a confirmatory study. Hence, metagenome from after-plow and vegetative stages were selected from each location in Kerala and compared to metagenomes of similar growth stages from locations separating thousands of miles apart in order to have a large variation in geography. India, being a sub-continent, has tremendous variation in geography, the climatic condition across such distance exhibits a considerable variation. Weighted UniFrac PCoA analysis showed clustering of samples based on geography (Figure 2B). However, an unweighted UniFrac PCoA plot clearly clustered the cropped stage irrespective of location (Figure 2A). The disparity between the PCoA plots could suggest that the microbial diversity in rice rhizosphere is influenced by the plant and remains relatively similar in spite of different location. However, their abundance is highly influenced by environmental factors due to the differential enrichment based on the environment. Similarly, unweighted UniFrac distances of maize rhizobiome had strong similarity deepened with plant age (Walters et al., 2018). In agreement to the PCoA, ADONIS analysis revealed location (*R*^2^ = 0.468 ± 0.049; *p* < 0.05) (Table 3) as the major determinant of the microbial community followed by plantation and growth stages. The strong influence of geography in shaping the community structure is in line with previous studies which state that the soil type and soil development stages had a major role compared to the plant effect (Mapelli et al., 2018). Further visualization of the dominant genera diversity and abundance (>1% abundance in any cultivation stage) in heatmap showed clear difference, albeit with some similarities, for each location, and cultivation stage (Supplementary Figure S6). Enriched dominant genera common to all location included *Ca*. Koribacter (nitrogen metabolism) (Júnior et al., 2019), *Ca*. Solibacter (nitrogen metabolism), *Clostridium* (nitrogen-fixing) (Doni et al., 2014), *Pseudonocardia* (siderophore production), *Geoalkalibacter* (syntrophic organic matter degradation) (Neveling et al., 2017), *Saccharopolyspora* (phosphate-mineralizing) (Franco-Correa and Chavarro-Anzola, 2016), and *Acidobacterium* (indole-3-acetic acid production) (Kielak et al., 2016b). Their prevalence could be because of the symbiotic association with the plants. For instance, in addition to IAA production, *Acidobacterium* spp. are able to reduce Fe^3+^ (ferric) to Fe^2+^ (ferrous) (Kielak et al., 2016b) which can be taken up by the plant.

### Rhizobiome Enrichment Is Strongly Linked to Plant Development

Abundance of ten top most abundant genera from each cultivation stage decreased during the cultivated stages as compared to uncultivated stage (pre-plow and post-harvest) (Figure 3A). The combined dominance of the top 10 most abundant genera during cropped stages reduced providing room for higher microbial diversity and complexity. *Ca.* Koribacter (14.61 ± 5.57%), *Ktedonobacter* (10.02 ± 3.56%), *Ca.* Solibacter (5.75 ± 2.51%), and *Peptoniphilus* (4.57 ± 1.68%) were among top 4 genera which consistently dominated in all the stages. Sharp differences in enrichment and depletion of the genera were prominent when the dominance was compared from one growth stage to another. *Ca.* Koribacter and *Ca.* Solibacter were enriched during the cropped stages while *Ktedonobacter* and *Peptoniphilus* were depleted. *Ca.* ndidatus *Koribacter* and *Ca.* ndidatus *Solibacter* possess large genomes with several gene duplications and paralogs obtained through horizontal gene transfer (Challacombe et al., 2011) and adapted to nutrient limited conditions (Eichorst et al., 2011), as well as to acidic soil (Grza̧dziel and Gała̧zka, 2018). High abundance of *Ca*. Koribacter and *Ca.* Solibacter were also reported in several soil (Navarrete et al., 2013; Júnior et al., 2019). However, their ecological functions is poorly understood due to lack of pure representative culture (Kielak et al., 2016a). Nonetheless, genomic insights have indicated that *Ca*. Koribacter and *Ca*. Solibacter are oligotrophic K-strategist have a strong role in reduction of nitrite and nitrate which helps in Nitrogen cycle (Fierer et al., 2007; Ward et al., 2009). *Ca*. Solibacter have a versatile genome which is adapted to breakdown complex recalcitrant organic compounds and carbohydrates and provides a suitable environmental condition for other microbes (Pearce et al., 2012; Rawat et al., 2012; Rime et al., 2015; Li et al., 2018). Previous reports have also highlighted their abundance in rhizosphere of black pepper (Umadevi et al., 2018), *Panax notoginseng* (Tan et al., 2017) and rice rhizosphere (Lopes et al., 2014). In line with previous report, *Ca.* Solibacter was positively correlated to rice cropping. *Ktedonobacteria* belonging to the Chloroflexi phylum were also highly abundant. However, their abundance depleted during cropped stages. *Ktedonobacter* were linked to tobacco disease since *Ktedonobacter* are non-nitrogen fixers which may compete with the microbial community for nitrogen source (Niu et al., 2016). However, in the pinus rhizosphere, *Ktedonobacter* were postulated to play a critical role in maintaining the microbial community structure (Wang et al., 2018). The genus *Peptoniphilus* under Firmicutes phylum was also found to decrease during cropped stages. *Peptoniphilus* has not been linked to plant rhizosphere to the best of our knowledge; however, there are several clinical reports of *Peptoniphilus* on human intestinal occlusion (Cobo et al., 2017), bloodstream infections (Brown et al., 2014), and various cancers (Shilnikova and Dmitrieva, 2015).

In order to study the patterns of genera having significant (*p* < 0.05) changes during cultivation stages, the rhizobiome enrichment in a stage wise manner was analyzed by identifying the entire genera which differed significantly (*p* < 0.05). A trend was observed in both enrichment as well as depleted microbes in a stage wise fashion (Figures 3C–I). Gradual enrichment was observed in the initial stages of cultivation followed by exponential increase between the vegetative and reproductive stage. This was followed by the formation of a plateau, which declined sharply after harvesting. This is expected since root exudates of rice as well as other plants have been documented to change according to plant maturity (Aulakh et al., 2001). Hence, the recruitment of beneficial microbes in the rhizosphere would vary as the plant matures. In addition, secondary metabolites from the rhizobiome aids in crosstalk with the microbes which further stimulates to secrete favorable exudates (Lareen et al., 2016). Such mutualistic relationship is necessary to avail several essential elements for plant development. For instance, bioavailability of phosphate in soil is critical for plant growth due to its role in morphological and physiological functions such as energy storage and transfer, cell division, root elongation, and photosynthesis, etc. (Yulianti and Rakhmawati, 2017). However, available forms of phosphate even in fertile soil is generally low at sub-micromolar levels (Arif et al., 2017). Phosphate solubilizing bacteria plays a critical role in solubilization of various phosphate compounds making it available to the plants (Arif et al., 2017), counteract soil calcification (Adnan et al., 2017), and enrich microbial diversity (Wang J. et al., 2017). Plant hormones are one of the major determinants of rooting and shooting (Habib et al., 2016), enhances drought resistance (Jung et al., 2015), up-regulates nitrogen fixation (Defez et al., 2017), and as bio-fertilizers (Shahzad et al., 2017). Such reprograming and cross talk between rhizobiome and plant leads to selective enrichment of beneficial microbes. Consistent with this, majority of the enriched dominant genera (*p* < 0.05, relative abundance > 0.1%) during the growth stages were PGPB involved in sulfur and nitrogen cycle as well as production of metabolites that aids in plant growth such as siderophore, IAA, ACC, phosphate solubilization, and antimicrobials. Similarly, further analysis between all the cropped stages vs. uncropped stages yield 143 genera (*p* < 0.05) (Supplementary Table S3) out of which 25 genera had relative abundance higher than 0.1% (Figure 3B). All the enriched genera (*n* = 19) related to IAA, ACC, siderophore, phosphate solubilization, etc. were represented during cropped stage while the suppressed genera (*n* = 6) were dominant in uncropped stage. Similar observations were made on soybean rhizosphere having higher abundance of plant growth promoting rhizobacteria in a stage dependent manner (Sugiyama et al., 2014). Enrichment of the rhizobiome was pronounced during the cropped stages and weaker during the off cultivation stages (Figure 3C). A strong correlation (*r* = 0.99; *p* = 0.02) was also observed between the number of enriched and suppressed genera during the growth stages. Previous studies have hypothesized that the high diversity and abundance of PGPB in the rhizosphere could decrease the non-PGPB due to the intense competition for resource and interference in the microbiome network.

### Aceticlastic Methanogens Dominate Rice Rhizosphere

Methane is a candidate for renewable energy source having high yield per mass unit (55.7 kJ/g) and burns cleaner than the fossil fuels (Richards et al., 2016). Hence, it holds a promising position in the energy industry. However, methane is 25 folds more potent greenhouse gas than CO_2_ (Yvon-Durocher et al., 2014). It is also the second most abundant greenhouse gas. Rice paddy contributes a significant amount of CH_4_ emission into the atmosphere (Smith et al., 2007) due to the ideal anoxic condition brought about by the prolonged waterlogged irrigation system. Methanogenesis is exclusively carried out by methanogens belonging to Archeal domain mainly by using H_2_/CO_2_ (cytochrome-lacking hydrogenotrophic methanogens) or acetate/methylated compounds as substrates (cytochrome-containing methylotrophic methanogens) (Vaksmaa et al., 2017). Methane emission in rice paddy is directly correlated to abundance of methanogen (Singh et al., 2017). Hence, in this study, microbial community of methanogens were investigated. Abundance of methanogens consistently declined until harvest (Figure 4A). Soil ammonium concentration analysis indicated an opposite trend to methanogens; ammonium concentration as well as AOA/AOB relative abundance increased with plant age (Figure 4C and Supplementary Figure S2). Nitrification produces NO_x_^–^ compounds which are partly taken up by the roots and the rest diffuses into the surroundings having adverse effect on methanogens (Banihani et al., 2009). In addition, methanogens require anaerobic condition for methanogensis. Hence, they are generally found with increased depth of 30–200 mm (Lee et al., 2015). We observed that the rice paddy methanogens mainly consisted of the *Methanosaeta*, *Methanocella*, and, *Methanobacterium* throughout the cultivation. Similar abundance has also been found in the rice paddy from South Korea, Philippine, Italy, and China (Lee et al., 2014; Breidenbach and Conrad, 2015; Hernández et al., 2015; Vaksmaa et al., 2017; Wang et al., 2018; Yuan et al., 2018). *Methanosaeta* was the most dominant methanogen in this study. It is capable of acetoclastic pathway for CH_4_ production and exhibits high affinity to acetate even at low concentration (Rotaru et al., 2014). Previous study from Indian rice paddy during cultivation has also reported the dominance of methanogenesis through acetoclastic pathway (Singh et al., 2012; Bhattacharyya et al., 2016). The second and third most abundance methanogen *Methanocella* and *Methanobacterium*, respectively, belongs to hydrogenotrophic methanogens. *Methanocella* belongs to the order Methanocellales which were formerly regarded as rice cluster I (RC-I) (Lü and Lu, 2012). They were found to produce CH_4_ from rice photosynthates (Lu and Conrad, 2005) and have adaption in low H_2_ environments (Liu et al., 2014).

### Type II Methanotrophs Are Less Diverse Yet More Abundant Than Type I

Methanotrophs resides in the rhizosphere owing to the sub-oxic zones created by the rice exudates. Methanotrophs are responsible for oxidizing at least half of the methane produced by methanogens before it is released to the atmosphere (Thauer, 2010). Hence they have been considered as a CH_4_ biological sink under aerobic as well as anaerobic condition (Leng et al., 2015). A strong pattern was observed in type I Methanotrophs being enriched with plant age until the drainage of logged water (Figure 4B). Similar to previous report from Indian rice paddy (Pandit et al., 2016b), a high diversity of type I methanotrophs were observed (Rahalkar et al., 2016; Pandit et al., 2018). Unlike type II Methanotrophs, type I methanotrophs have atmospheric nitrogen fixing capability and derive nitrogen from atmospheric N in the absence of other N sources (Noll et al., 2008). Hence, selective enrichment by root exudates could establish a symbiotic relationship due to the nitrogen fixing properties of type I Methanotrophs. However, genus *Methylocystis* (type II methanotrophs) was the most abundant among the methanotrophs (Figure 4B). Various other studies have observed similar dominance of *Methylocystis* in India (Pandit et al., 2016a), China (Liu et al., 2017), Taiwan (Shiau et al., 2018), and South Korea (Lee et al., 2014). This could be due to its survival physiology in dry environment during the off season by forming desiccation-resistant cysts (Pandit et al., 2016a). It can also be attributed to the dual nature of *Methylocystis* to obtain carbon from methane as well as acetate (Leng et al., 2015). On a broader view, similar to previous studies (Lee et al., 2014), type II-Methanotrophs didn’t show any significant enrichment with plant age. *In vitro* studies by have shown that type II Methanotrophs were inhibited in paddy soils by ammonium treatment possibly due to the substrate competition between methane monooxygenase and ammonia monooxygenase (Reay and Nedwell, 2004) and also by the toxicity of ammonia oxidation byproducts such as hydroxylamine and nitrite (Saari et al., 2004). However, the negative effect of ammonia could also be counteracted to some extent by the end product of ammonia oxidation, nitrate, which stimulates the growth of both type I, and type II Methanotrophs (Hu and Lu, 2015). Genus *Methylacidiphilum* under phylum Verrucomicrobia was found throughout the cultivation (Figure 4B). *Methylacidiphilum* have also been recently reported in Italian paddy soil (Vaksmaa et al., 2017). This genus is of special interest because they are the most acidophilic methanotrophs which was originally discovered from a geothermal ecosystem (Op den Camp et al., 2009). They can thrive at pH 2 (Islam et al., 2008) and found mostly in extreme conditions such as geothermal environment and volcanic soil (van Teeseling et al., 2014). Its presence in paddy soil hints toward the need to understand its role in the rice paddy methane oxidation (Hou et al., 2008). Overall, type I methanotrophs showed a higher diversity (*n* = 12) than type II Methanotrophs (*n* = 6) yet type II methanotrophs had a significantly higher abundance (*p* = 0.003).

### AOA Abundance Dominates Over AOB in Flooded Rice Paddy

AOA was represented several folds higher than AOB in all the cultivation stages (Figure 4C). In line to our observation, meta-analysis of East Asian paddy soils have found similar dominance of AOA over AOB in acidic as well as alkaline paddy soils (Mukhtar et al., 2017). AOA have been shown to be highly adaptive in microaerophilic condition (Wang et al., 2015) and greatly influence by the rice root exudates (Chen et al., 2008). We found that within AOA community, Nitrosocaldus cluster dominated the paddy soil. Nitrosophaera cluster was the 2nd most dominant group in our study. However, Nitrosophaera cluster was the most dominated within AOA community in several studies from China (Chen et al., 2008; Ke et al., 2013; Wang et al., 2015; Lu et al., 2018) and East Asian paddy soils meta-analysis (Mukhtar et al., 2017). The disparity between our study and literature could be attributed to the major difference in PCR primer. Previous studies have implemented the widely used Archaeal *amoA* gene primers which has several mismatch base pairs to Nitrosocaldus cluster (Ke et al., 2013). Hence, comparative analysis of amoA based literature should be taken with precaution. As for AOB community, Nitrosospira was the most dominant. Similar observation were found in alkaline as well as neutral paddy soil from China (Chen et al., 2008; Lu et al., 2018), Japan (Bowatte et al., 2006), East Asian paddy soils meta-analysis (Mukhtar et al., 2017). AOA has been found to be dominating in acidic soils (pH < 5.5) (Liu et al., 2013). They are also highly adaptive to low ammonia concentration having highest affinity toward ammonia among all ammonia oxidizers and tolerate sub-oxic condition created in the rhizosphere through the oxygen leakage from root surface and diffusion of atmospheric oxygen (Wang et al., 2015). In addition, AOA has also been reported to thrive in wet environment (Hu et al., 2015). Corroborating to this, AOA abundance increased sharply after-plow which declined after drainage during pre-harvest stage indicating that the flooded water irrigation system favors AOA. *Candidatus Nitrosocaldus* genus (AOA) was the most dominant among all ammonia oxidizers. AOB abundance increased gradually with plant growth till pre-harvest stage (Figure 4C). Hence, AOB were less affected by the change in the flooded condition. AOB are known to thrive in high ammonium concentration (Jia and Conrad, 2009). Within AOB, *Nitrosospira* was the most dominant and follows a K strategy having high affinity to low ammonia concentration (Zhang et al., 2016). Overall the diversity of AOA (*n* = 3) as well as AOB (*n* = 4) was low. Previous study (Han et al., 2018) has noted that the diversity of ammonia oxidizers is minimum in long-term water input. Since the sampled geographical location experiences wet tropical climate, the low diversity of ammonia oxidizers is expected. Within the nitrite oxidizing bacteria, *Nitrospira* genus was found to be most abundant (Figure 4D). *Nitrospira* play a major role in terrestrial ecosystems and withstand high concentration of nitrite (Han et al., 2018). *Nitrospira* are fast-growing K-strategists having high affinity to substrate even at low nitrite and oxygen concentrations having resistance to ampicillin and Acriflavine. It has been postulated that the high efficiency of *Candidatus Nitrospira defluvii* could be due to its unique presence (found only in *Nitrospira*, *Nitrospina*, and *Anammox* bacteria) of nitrite oxidoreductase, a key enzyme in NO_2_^–^ oxidation, in periplasmic region rather than cytoplasm. This orientation favors the generation of more proton-motive force per nitrate oxidized (Rodríguez et al., 2015).

## Conclusion

16S rRNA gene amplicon based metagenomic analysis of rice bulk soil and rhizobiome was carried out at different cultivation stages showing sharp distinction in the alpha diversity and enrichment as the plant matured. The rich network complexity during the plantation points out the enormous diversity of microbes, which can be harnessed not only for improved plant health as bio-fertilizer but as a reservoir of novel enzymes and metabolites yet to be explored. Looking at an ecological point of view, it is counterintuitive to find that the methanotrophs declined with plant growth in the rhizobiome. However, methanogensis at the higher depth rhizosphere would be interesting to investigate further in Indian paddy rice irrigation system. In addition, the higher abundance of AOA over AOB further adds to the evidence that AOA dominates the ammonia oxidation in a majority of ecosystems.

Although the analysis of microbial community through 16S rRNA gene amplicon based metagenomic is a powerful tool and provides a significantly larger view as compared to DGGE or traditional culture based techniques. However, it comes with certain limitation. Firstly, such technique relies on the relative abundance rather than absolute abundance of the microbial community. Recent reports as per Naylor et al. (2017) and Fitzpatrick et al. (2018) have indicated that the relative abundance from such high throughput data could reflect the absolute abundance fluctuation. Secondly, the functional potential of the microbial community analyzed based on genera could have multiple outcomes based on the species and strains. Thirdly, 16S rRNA gene amplicon based metagenomic relies on the use of primers for amplification of the 16S rRNA gene, which could introduce biases. However, Ibarbalz et al. (2014) reported that despite the 16S rRNA gene amplification bias, it provides a strong quantitative and qualitative information of the microbial community yet the archeal community such as methanotrophs, methanogens, and ammonia oxidizers could be under represented (Raymann et al., 2017).

## Data Availability

The raw fastQ files for the Kerala, India samples were uploaded in MG-RAST server and publicly available from the MG-RAST server under IDs mgm4758099.3 to mgm4758122.3.

## Author Contributions

RK and MI conceived and designed the experiments. RK, MI, DB, and AV analyzed the data. RK contributed reagents, materials, and analysis tools. RK, MI, AV, AG-N, NK, OP, DB, PG, and VA wrote the manuscript.

## Conflict of Interest Statement

The authors declare that the research was conducted in the absence of any commercial or financial relationships that could be construed as a potential conflict of interest.

## References

[B1] Adnan M. Shah Z. Fahad S. Arif M. Alam M. Khan I. A. (2017). Phosphate-solubilizing bacteria nullify the antagonistic effect of soil calcification on bioavailability of phosphorus in alkaline soils. *Sci. Rep.* 7:16131. 10.1038/s41598-017-16537-5 29170494 PMC5701022

[B2] Arif M. S. Shahzad S. M. Yasmeen T. Riaz M. Ashraf M. Ashraf M. A. (2017). “Improving plant phosphorus (P) acquisition by phosphate-solubilizing bacteria,” in *Essential Plant Nutrients*, eds Naeem M. Ansari A. Gill S. (Cham: Springer), 513–556. 10.1007/978-3-319-58841-4_21

[B3] Aulakh M. S. Wassmann R. Bueno C. Kreuzwieser J. Rennenberg H. (2001). Characterization of root exudates at different growth stages of ten rice (*Oryza sativa L*.) cultivars. *Plant Biol.* 3 139–148. 10.1055/s-2001-12905

[B4] Banihani Q. Sierra-Alvarez R. Field J. A. (2009). Nitrate and nitrite inhibition of methanogenesis during denitrification in granular biofilms and digested domestic sludges. *Biodegradation* 20 801–812. 10.1007/s10532-009-9268-9 19449209

[B5] Bastian M. Heymann S. Jacomy M. (2009). “Gephi: an open source software for exploring and manipulating networks,” in *Proceedings of the 3rd International AAAI Conference on Weblogs and Social Media*, San Jose, CA.

[B6] Bhattacharyya P. Roy K. S. Das M. Ray S. Balachandar D. Karthikeyan S. (2016). Elucidation of rice rhizosphere metagenome in relation to methane and nitrogen metabolism under elevated carbon dioxide and temperature using whole genome metagenomic approach. *Sci. Total Environ.* 542 886–898. 10.1016/j.scitotenv.2015.10.154 26556753

[B7] Bowatte S. Jia Z. Ishihara R. Nakajima Y. Asakawa S. Kimura M. (2006). Molecular analysis of the ammonia oxidizing bacterial community in the surface soil layer of a Japanese paddy field. *Soil Sci. Plant Nutr.* 52 427–431. 10.1111/j.1747-0765.2006.00058.x

[B8] Breidenbach B. Conrad R. (2015). Seasonal dynamics of bacterial and archaeal methanogenic communities in flooded rice fields and effect of drainage. *Front. Microbiol.* 5:752. 10.3389/fmicb.2014.00752 25620960 PMC4288041

[B9] Brown K. Church D. Lynch T. Gregson D. (2014). Bloodstream infections due to *P eptoniphilus* spp.: report of 15 cases. *Clin. Microbiol. Infect.* 20 O857–O860. 10.1111/1469-0691.12657 24773457 PMC4304329

[B10] Challacombe J. F. Eichorst S. A. Hauser L. Land M. Xie G. Kuske C. R. (2011). Biological consequences of ancient gene acquisition and duplication in the large genome of *Candidatus Solibacter usitatus* Ellin6076. *PLoS One* 6:e24882. 10.1371/journal.pone.0024882 21949776 PMC3174227

[B11] Chaparro J. M. Badri D. V. Vivanco J. M. (2014). Rhizosphere microbiome assemblage is affected by plant development. *ISME J.* 8 790–803. 10.1038/ismej.2013.196 24196324 PMC3960538

[B12] Chen X. P. Zhu Y. G. Xia Y. Shen J. P. He J. Z. (2008). Ammonia-oxidizing archaea: important players in paddy rhizosphere soil? *Environ. Microbiol.* 10 1978–1987. 10.1111/j.1462-2920.2008.01613.x 18430011

[B13] Cobo F. Rodríguez-Granger J. Sampedro A. Navarro-Marí J. M. (2017). Peritoneal infection due to *Peptoniphilus harei* in a patient with intestinal occlusion. *Anaerobe* 44 126–127. 10.1016/j.anaerobe.2017.03.009 28286023

[B14] Cox M. P. Peterson D. A. Biggs P. J. (2010). SolexaQA: at-a-glance quality assessment of Illumina second-generation sequencing data. *BMC Bioinform.* 11:485. 10.1186/1471-2105-11-485 20875133 PMC2956736

[B15] Defez R. Andreozzi A. Bianco C. (2017). The overproduction of indole-3-acetic acid (IAA) in endophytes upregulates nitrogen fixation in both bacterial cultures and inoculated rice plants. *Microb. Ecol.* 74 441–452. 10.1007/s00248-017-0948-4 28197647

[B16] Doni F. Anizan I. CheRadziah C. M. Z. Wan Natasya W. A. Abidah A. Suryadi E. (2014). Enhanced rice seedling growth by Clostridium and *Pseudomonas*. *Biotechnology* 13 186–189. 10.3923/biotech.2014.186.189

[B17] Donn S. Kirkegaard J. A. Perera G. Richardson A. E. Watt M. (2015). Evolution of bacterial communities in the wheat crop rhizosphere. *Environ. Microbiol.* 17 610–621. 10.1111/1462-2920.12452 24628845

[B18] Duineveld B. M. Kowalchuk G. A. Keijzer A. van Elsas J. D. van Veen J. A. (2001). Analysis of bacterial communities in the rhizosphere of chrysanthemum via denaturing gradient gel electrophoresis of PCR-amplified 16S rRNA as well as DNA fragments coding for 16S rRNA. *Appl. Environ. Microbiol.* 67 172–178. 10.1128/aem.67.1.172-178.2001 11133442 PMC92540

[B19] Edwards J. Johnson C. Santos-Medellín C. Lurie E. Podishetty N. K. Bhatnagar S. (2015). Structure, variation, and assembly of the root-associated microbiomes of rice. *Proc. Natl. Acad. Sci. U.S.A.* 112 E911–E920. 10.1073/pnas.1414592112 25605935 PMC4345613

[B20] Eichorst S. A. Kuske C. R. Schmidt T. M. (2011). Influence of plant polymers on the distribution and cultivation of bacteria in the phylum Acidobacteria. *Appl. Environ. Microbiol.* 77 586–596. 10.1128/AEM.01080-10 21097594 PMC3020536

[B21] Fierer N. Bradford M. A. Jackson R. B. (2007). Toward an ecological classification of soil bacteria. *Ecology* 88 1354–1364. 10.1890/05-1839 17601128

[B22] Fierer N. Jackson R. B. (2006). The diversity and biogeography of soil bacterial communities. *Proc. Natl. Acad. Sci. U.S.A.* 103 626–631. 10.1073/pnas.0507535103 16407148 PMC1334650

[B23] Fitzpatrick C. R. Copeland J. Wang P. W. Guttman D. S. Kotanen P. M. Johnson M. T. (2018). Assembly and ecological function of the root microbiome across angiosperm plant species. *Proc. Natl. Acad. Sci. U.S.A.* 115 E1157–E1165. 10.1073/pnas.1717617115 29358405 PMC5819437

[B24] Franco-Correa M. Chavarro-Anzola V. (2016). “Actinobacteria as plant growth promoting rhizobacteria,” in *Actinobacteria-Basis and Biotechnological Application*, eds Dhanasekaran D. Jiang Y. (London: InTechOpen, 249–270.

[B25] Gomez-Alvarez V. Teal T. K. Schmidt T. M. (2009). Systematic artifacts in metagenomes from complex microbial communities. *ISME J.* 3 1314–1317. 10.1038/ismej.2009.72 19587772

[B26] Gonzalez-Franco A. C. Robles-Hernandez L. Nuñez-Barrios A. Strap J. L. Crawford D. L. (2009). Molecular and cultural analysis of seasonal actinomycetes in soils from *Artemisia tridentata* habitat. *Phyton* 78 83–90.

[B27] Grza̧dziel J. Gała̧zka A. (2018). Microplot long-term experiment reveals strong soil type influence on bacteria composition and its functional diversity. *Appl. Soil Ecol.* 124 117–123. 10.1016/j.apsoil.2017.10.033

[B28] Habib S. H. Kausar H. Saud H. M. Ismail M. R. Othman R. (2016). Molecular characterization of stress tolerant plant growth promoting rhizobacteria (PGPR) for growth enhancement of rice. *Int. J. Agric. Biol.* 18 184–191. 10.17957/ijab/15.0094

[B29] Han S. Li X. Luo X. Wen S. Chen W. Huang Q. (2018). Nitrite-oxidizing bacteria community composition and diversity are influenced by fertilizer regimes, but are independent of the soil aggregate in acidic subtropical red soil. *Front. Microbiol.* 9:885. 10.3389/fmicb.2018.00885 29867799 PMC5951965

[B30] Hartmann A. Rothballer M. Schmid M. (2008). Lorenz Hiltner, a pioneer in rhizosphere microbial ecology and soil bacteriology research. *Plant Soil* 312 7–14. 10.1007/s11104-007-9514-z

[B31] He S. Guo L. Niu M. Miao F. Jiao S. Hu T. (2017). Ecological diversity and co-occurrence patterns of bacterial community through soil profile in response to long-term switchgrass cultivation. *Sci. Rep.* 7:3608. 10.1038/s41598-017-03778-7 28620188 PMC5472595

[B32] Hernández M. Dumont M. G. Yuan Q. Conrad R. (2015). Different bacterial populations associated with the roots and rhizosphere of rice incorporate plant-derived carbon. *Appl. Environ. Microbiol.* 81 2244–2253. 10.1128/AEM.03209-14 25616793 PMC4345361

[B33] Hou S. Makarova K. S. Saw J. H. Senin P. Ly B. V. Zhou Z. (2008). Complete genome sequence of the extremely acidophilic methanotroph isolate V4, *Methylacidiphilum infernorum*, a representative of the bacterial phylum Verrucomicrobia. *Biol. Direct* 3:26. 10.1186/1745-6150-3-26 18593465 PMC2474590

[B34] Hu A. Lu Y. (2015). The differential effects of ammonium and nitrate on methanotrophs in rice field soil. *Soil Biol. Biochem.* 85 31–38. 10.1016/j.soilbio.2015.02.033

[B35] Hu H. W. Macdonald C. A. Trivedi P. Holmes B. Bodrossy L. He J. Z. (2015). Water addition regulates the metabolic activity of ammonia oxidizers responding to environmental perturbations in dry subhumid ecosystems. *Environ. Microbiol.* 17 444–461. 10.1111/1462-2920.12481 24725346

[B36] Ibarbalz F. M. Pérez M. V. Figuerola E. L. Erijman L. (2014). The bias associated with amplicon sequencing does not affect the quantitative assessment of bacterial community dynamics. *PLoS One* 9:e99722. 10.1371/journal.pone.0099722 24923665 PMC4055690

[B37] Innes L. Hobbs P. J. Bardgett R. D. (2004). The impacts of individual plant species on rhizosphere microbial communities in soils of different fertility. *Biol. Fertil. Soils* 40 7–13. 10.1007/s00374-004-0748-0

[B38] Islam T. Jensen S. Reigstad L. J. Larsen Ø Birkeland N. K. (2008). Methane oxidation at 55 C and pH 2 by a thermoacidophilic bacterium belonging to the Verrucomicrobia phylum. *Proc. Natl. Acad. Sci. U.S.A.* 105 300–304. 10.1073/pnas.0704162105 18172218 PMC2224206

[B39] Jia Z. Conrad R. (2009). Bacteria rather than Archaea dominate microbial ammonia oxidation in an agricultural soil. *Environ. Microbiol.* 11 1658–1671. 10.1111/j.1462-2920.2009.01891.x 19236445

[B40] Jung H. Lee D. K. Do Choi Y. Kim J. K. (2015). OsIAA6, a member of the rice Aux/IAA gene family, is involved in drought tolerance and tiller outgrowth. *Plant Sci.* 236 304–312. 10.1016/j.plantsci.2015.04.018 26025543

[B41] Júnior L. Vieira G. Noronha M. F. Cabral L. Delforno T. P. de Sousa S. T. P. (2019). Land use and seasonal effects on the soil microbiome of a Brazilian dry forest. *Front. Microbiol.* 10:648. 10.3389/fmicb.2019.00648 31024471 PMC6461016

[B42] Ke X. Angel R. Lu Y. Conrad R. (2013). Niche differentiation of ammonia oxidizers and nitrite oxidizers in rice paddy soil. *Environ. Microbiol.* 15 2275–2292. 10.1111/1462-2920.12098 23437806

[B43] Kielak A. M. Barreto C. C. Kowalchuk G. A. van Veen J. A. Kuramae E. E. (2016a). The ecology of acidobacteria: moving beyond genes and genomes. *Front. Microbiol.* 7:744. 10.3389/fmicb.2016.00744 27303369 PMC4885859

[B44] Kielak A. M. Cipriano M. A. Kuramae E. E. (2016b). Acidobacteria strains from subdivision 1 act as plant growth-promoting bacteria. *Arch. Microbiol.* 198 987–993. 10.1007/s00203-016-1260-2 27339258 PMC5080364

[B45] Klotz M. G. Stein L. Y. (2011). *Research on Nitrification and Related Processes*, Vol. 496 Cambridge, MA: Academic Press. 10.1016/B978-0-12-386489-5.00027-0 21514457

[B46] Lareen A. Burton F. Schäfer P. (2016). Plant root-microbe communication in shaping root microbiomes. *Plant Mol. Biol.* 90 575–587. 10.1007/s11103-015-0417-8 26729479 PMC4819777

[B47] Lee H. J. Jeong S. E. Kim P. J. Madsen E. L. Jeon C. O. (2015). High resolution depth distribution of Bacteria, Archaea, methanotrophs, and methanogens in the bulk and rhizosphere soils of a flooded rice paddy. *Front. Microbiol.* 6:639. 10.3389/fmicb.2015.00639 26161079 PMC4479796

[B48] Lee H. J. Kim S. Y. Kim P. J. Madsen E. L. Jeon C. O. (2014). Methane emission and dynamics of methanotrophic and methanogenic communities in a flooded rice field ecosystem. *FEMS Microbiol. Ecol.* 88 195–212. 10.1111/1574-6941.12282 24410836

[B49] Leng L. Chang J. Geng K. Lu Y. Ma K. (2015). Uncultivated *Methylocystis* species in paddy soil include facultative methanotrophs that utilize acetate. *Microb. Ecol.* 70 88–96. 10.1007/s00248-014-0540-0 25475784

[B50] Li Q. Liu C. Wang X. Jin Z. Song A. Liang Y. (2018). Influence of altered microbes on soil organic carbon availability in karst agricultural soils contaminated by Pb-Zn tailings. *Front. Microbiol.* 9:2062. 10.3389/fmicb.2018.02062 30233539 PMC6127319

[B51] Liu J. Xu H. Jiang Y. Zhang K. Hu Y. Zeng Z. (2017). Methane emissions and microbial communities as influenced by dual cropping of Azolla along with early rice. *Sci. Rep.* 7:40635. 10.1038/srep40635 28094773 PMC5240575

[B52] Liu K. McInroy J. A. Hu C. H. Kloepper J. W. (2018). Mixtures of plant-growth-promoting rhizobacteria enhance biological control of multiple plant diseases and plant-growth promotion in the presence of pathogens. *Plant Dis.* 102 67–72. 10.1094/PDIS-04-17-0478-RE 30673446

[B53] Liu P. Yang Y. Lü Z. Lu Y. (2014). Response of a rice paddy soil methanogen to syntrophic growth as revealed by transcriptional analyses. *Appl. Environ. Microbiol.* 80 4668–4676. 10.1128/aem.01259-14 24837392 PMC4148802

[B54] Liu S. Shen L. Lou L. Tian G. Zheng P. Hu B. (2013). Spatial distribution and factors shaping the niche segregation of ammonia-oxidizing microorganisms in the Qiantang River, China. *Appl. Environ. Microbiol.* 79 4065–4071. 10.1128/AEM.00543-13 23624482 PMC3697550

[B55] Lopes A. R. Manaia C. M. Nunes O. C. (2014). Bacterial community variations in an alfalfa-rice rotation system revealed by 16S rRNA gene 454-pyrosequencing. *FEMS Microbiol. Ecol.* 87 650–663. 10.1111/1574-6941.12253 24245591

[B56] Lu L. Li H. He Y. Zhang J. Xiao J. Peng C. (2018). Compositional shifts in ammonia-oxidizing microorganism communities of eight geographically different paddy soils. *Agric. Sci.* 9 351–373. 10.4236/as.2018.93025

[B57] Lu Y. Conrad R. (2005). In situ stable isotope probing of methanogenic archaea in the rice rhizosphere. *Science* 309 1088–1090. 10.1126/science.1113435 16099988

[B58] Lü Z. Lu Y. (2012). *Methanocella conradii* sp. nov., a thermophilic, obligate hydrogenotrophic methanogen, isolated from Chinese rice field soil. *PLoS One* 7:e35279. 10.1371/journal.pone.0035279 22530002 PMC3328440

[B59] Mapelli F. Marasco R. Fusi M. Scaglia B. Tsiamis G. Rolli E. (2018). The stage of soil development modulates rhizosphere effect along a High Arctic desert chronosequence. *ISME J.* 12 1188–1198. 10.1038/s41396-017-0026-4 29335640 PMC5931989

[B60] Mendes L. W. Kuramae E. E. Navarrete A. A. Van Veen J. A. Tsai S. M. (2014). Taxonomical and functional microbial community selection in soybean rhizosphere. *ISME J.* 8 1577–1587. 10.1038/ismej.2014.17 24553468 PMC4817605

[B61] Mendes R. Garbeva P. Raaijmakers J. M. (2013). The rhizosphere microbiome: significance of plant beneficial, plant pathogenic, and human pathogenic microorganisms. *FEMS Microbiol. Rev.* 37 634–663. 10.1111/1574-6976.12028 23790204

[B62] Meyer F. Paarmann D. D’Souza M. Olson R. Glass E. M. Kubal M. (2008). The metagenomics RAST server–a public resource for the automatic phylogenetic and functional analysis of metagenomes. *BMC Bioinform.* 9:386. 10.1186/1471-2105-9-386 18803844 PMC2563014

[B63] Mukhtar H. Lin Y. P. Anthony J. (2017). Ammonia oxidizing archaea and bacteria in east Asian paddy soils—a mini review. *Environments* 4:84 10.3390/environments4040084

[B64] Muthayya S. Sugimoto J. D. Montgomery S. Maberly G. F. (2014). An overview of global rice production, supply, trade, and consumption. *Ann. N. Y. Acad. Sci.* 1324 7–14. 10.1111/nyas.12540 25224455

[B65] Navarrete A. A. Kuramae E. E. de Hollander M. Pijl A. S. van Veen J. A. Tsai S. M. (2013). Acidobacterial community responses to agricultural management of soybean in Amazon forest soils. *FEMS Microbiol. Ecol.* 83 607–621. 10.1111/1574-6941.12018 23013447

[B66] Naylor D. DeGraaf S. Purdom E. Coleman-Derr D. (2017). Drought and host selection influence bacterial community dynamics in the grass root microbiome. *ISME J.* 11 2691–2704. 10.1038/ismej.2017.118 28753209 PMC5702725

[B67] Neue H. U. (1993). Methane emission from rice fields. *Bioscience* 43 466–474. 10.2307/1311906

[B68] Neveling D. P. Van Emmenes L. Ahire J. J. Pieterse E. Smith C. Dicks L. M. T. (2017). Safety assessment of antibiotic and probiotic feed additives for *Gallus gallus domesticus*. *Sci. Rep.* 7:12767. 10.1038/s41598-017-12866-7 29038560 PMC5643334

[B69] Niu J. Rang Z. Zhang C. Chen W. Tian F. Yin H. (2016). The succession pattern of soil microbial communities and its relationship with tobacco bacterial wilt. *BMC Microbiol.* 16:233. 10.1186/s12866-016-0845-x 27716043 PMC5054579

[B70] Noll M. Frenzel P. Conrad R. (2008). Selective stimulation of type I methanotrophs in a rice paddy soil by urea fertilization revealed by RNA-based stable isotope probing. *FEMS Microbiol. Ecol.* 65 125–132. 10.1111/j.1574-6941.2008.00497.x 18544098

[B71] Novello G. Gamalero E. Bona E. Boatti L. Mignone F. Massa N. (2017). The rhizosphere bacterial microbiota of *Vitis vinifera* cv. pinot noir in an integrated pest management vineyard. *Front. Microbiol.* 8:1528. 10.3389/fmicb.2017.01528 28855895 PMC5557794

[B72] Op den Camp H. J. Islam T. Stott M. B. Harhangi H. R. Hynes A. Schouten S. (2009). Environmental, genomic and taxonomic perspectives on methanotrophic Verrucomicrobia. *Environ. Microbiol. Rep.* 1 293–306. 10.1111/j.1758-2229.2009.00022.x 23765882

[B73] Pandit P. S. Hoppert M. Rahalkar M. C. (2018). Description of ‘Candidatus Methylocucumis oryzae’, a novel Type I methanotroph with large cells and pale pink colour, isolated from an Indian rice field. *Antonie Van Leeuwenhoek* 111 2473–2484. 10.1007/s10482-018-1136-3 30066210

[B74] Pandit P. S. Rahalkar M. C. Dhakephalkar P. K. Ranade D. R. Pore S. Arora P. (2016a). Deciphering community structure of methanotrophs dwelling in rice rhizospheres of an Indian rice field using cultivation and cultivation-independent approaches. *Microb. Ecol.* 71 634–644. 10.1007/s00248-015-0697-1 26547567

[B75] Pandit P. S. Ranade D. R. Dhakephalkar P. K. Rahalkar M. C. (2016b). A pmoA-based study reveals dominance of yet uncultured Type I methanotrophs in rhizospheres of an organically fertilized rice field in India. *3 Biotech* 6:135. 10.1007/s13205-016-0453-3 28330207 PMC4910840

[B76] Pearce D. A. Newsham K. K. Thorne M. A. Calvo-Bado L. Krsek M. Laskaris P. (2012). Metagenomic analysis of a southern maritime Antarctic soil. *Front. Microbiol.* 3:403 10.3389/fmicb.2012.00403 PMC3514609 23227023

[B77] Peiffer J. A. Spor A. Koren O. Jin Z. Tringe S. G. Dangl J. L. (2013). Diversity and heritability of the maize rhizosphere microbiome under field conditions. *Proc. Natl. Acad. Sci. U.S.A.* 110 6548–6553. 10.1073/pnas.1302837110 23576752 PMC3631645

[B78] Philippot L. Raaijmakers J. M. Lemanceau P. Van Der Putten W. H. (2013). Going back to the roots: the microbial ecology of the rhizosphere. *Nat. Rev. Microbiol.* 11 789–799. 10.1038/nrmicro3109 24056930

[B79] Pittol M. Scully E. Miller D. Durso L. Mariana Fiuza L. Valiati V. H. (2018). Bacterial community of the rice floodwater using cultivation-independent approaches. *Int. J. Microbiol.* 2018:6280484. 10.1155/2018/6280484 29666650 PMC5831270

[B80] Qiao Q. Wang F. Zhang J. Chen Y. Zhang C. Liu G. (2017). The variation in the rhizosphere microbiome of cotton with soil type, genotype and developmental stage. *Sci. Rep.* 7:3940. 10.1038/s41598-017-04213-7 28638057 PMC5479781

[B81] R Core Team (2014). *R Foundation for Statistical Computing.* Vienna: R Core Team.

[B82] Rahalkar M. C. Pandit P. S. Dhakephalkar P. K. Pore S. Arora P. Kapse N. (2016). Genome characteristics of a novel type I methanotroph (Sn10-6) isolated from a flooded Indian rice field. *Microb. Ecol.* 71 519–523. 10.1007/s00248-015-0699-z 26547566

[B83] Rawat S. R. Männistö M. K. Bromberg Y. Häggblom M. M. (2012). Comparative genomic and physiological analysis provides insights into the role of Acidobacteria in organic carbon utilization in Arctic tundra soils. *FEMS Microbiol. Ecol.* 82 341–355. 10.1111/j.1574-6941.2012.01381.x 22486608

[B84] Raymann K. Moeller A. H. Goodman A. L. Ochman H. (2017). Unexplored archaeal diversity in the great ape gut microbiome. *mSphere* 2:e00026-17. 10.1128/mSphere.00026-17 28251182 PMC5322346

[B85] Reay D. S. Nedwell D. B. (2004). Methane oxidation in temperate soils: effects of inorganic N. *Soil Biol. Biochem.* 36 2059–2065. 10.1016/j.soilbio.2004.06.002

[B86] Richards M. A. Lie T. J. Zhang J. Ragsdale S. W. Leigh J. A. Price N. D. (2016). Exploring hydrogenotrophic methanogenesis: a genome scale metabolic reconstruction of *Methanococcus maripaludis*. *J. Bacteriol.* 198 3379–3390. 10.1128/jb.00571-16 27736793 PMC5116941

[B87] Rime T. Hartmann M. Brunner I. Widmer F. Zeyer J. Frey B. (2015). Vertical distribution of the soil microbiota along a successional gradient in a glacier forefield. *Mol. Ecol.* 24 1091–1108. 10.1111/mec.13051 25533315

[B88] Rodríguez E. García-Encina P. A. Stams A. J. Maphosa F. Sousa D. Z. (2015). Meta-omics approaches to understand and improve wastewater treatment systems. *Rev. Environ. Sci. BioTechnol.* 14 385–406. 10.1007/s11157-015-9370-x

[B89] Rotaru A. E. Shrestha P. M. Liu F. Shrestha M. Shrestha D. Embree M. (2014). A new model for electron flow during anaerobic digestion: direct interspecies electron transfer to *Methanosaeta* for the reduction of carbon dioxide to methane. *Energy Environ. Sci.* 7 408–415. 10.1039/c3ee42189a

[B90] Saari A. Rinnan R. Martikainen P. J. (2004). Methane oxidation in boreal forest soils: kinetics and sensitivity to pH and ammonium. *Soil Biol. Biochem.* 36 1037–1046. 10.1016/j.soilbio.2004.01.018

[B91] Schnitzer S. A. Klironomos J. N. HilleRisLambers J. Kinkel L. L. Reich P. B. Xiao K. (2011). Soil microbes drive the classic plant diversity–productivity pattern. *Ecology* 92 296–303. 10.1890/10-0773.1 21618909

[B92] Shahzad R. Waqas M. Khan A. L. Al-Hosni K. Kang S. M. Seo C. W. (2017). Indoleacetic acid production and plant growth promoting potential of bacterial endophytes isolated from rice (*Oryza sativa* L.) seeds. *Acta Biol. Hungarica* 68 175–186. 10.1556/018.68.2017.2.5 28605980

[B93] Shi S. Nuccio E. Herman D. J. Rijkers R. Estera K. Li J. (2015). Successional trajectories of rhizosphere bacterial communities over consecutive seasons. *mBio* 6:e00746. 10.1128/mBio.00746-15 26242625 PMC4526712

[B94] Shi S. Nuccio E. E. Shi Z. J. He Z. Zhou J. Firestone M. K. (2016). The interconnected rhizosphere: high network complexity dominates rhizosphere assemblages. *Ecol. Lett.* 19 926–936. 10.1111/ele.12630 27264635

[B95] Shiau Y. J. Cai Y. Jia Z. Chen C. L. Chiu C. Y. (2018). Phylogenetically distinct methanotrophs modulate methane oxidation in rice paddies across Taiwan. *Soil Biol. Biochem.* 124 59–69. 10.1016/j.soilbio.2018.05.025

[B96] Shilnikova I. I. Dmitrieva N. V. (2015). Evaluation of antibiotic susceptibility of gram-positive anaerobic cocci isolated from cancer patients of the NN Blokhin Russian cancer research center. *J. Pathog.* 2015:648134. 10.1155/2015/648134 PMC4698780 26798518

[B97] Singh A. Singh R. S. Upadhyay S. N. Joshi C. G. Tripathi A. K. Dubey S. K. (2012). Community structure of methanogenic archaea and methane production associated with compost-treated tropical rice-field soil. *FEMS Microbiol. Ecol.* 82 118–134. 10.1111/j.1574-6941.2012.01411.x 22587395

[B98] Singh A. Srivastva N. Dubey S. K. (2017). Molecular diversity of methanogenic archaea and methane production potential of soil in relation to rice cultivars. *Indian J. Exp. Biol.* 55 506–513.

[B99] Smith P. Martino D. Cai Z. Gwary D. Janzen H. Kumar P. (2007). Policy and technological constraints to implementation of greenhouse gas mitigation options in agriculture. *Agric. Ecosyst. Environ.* 118 6–28. 10.1016/j.agee.2006.06.006

[B100] Sugiyama A. Ueda Y. Zushi T. Takase H. Yazaki K. (2014). Changes in the bacterial community of soybean rhizospheres during growth in the field. *PLoS One* 9:e100709. 10.1371/journal.pone.0100709 24955843 PMC4067361

[B101] Takahashi S. Tomita J. Nishioka K. Hisada T. Nishijima M. (2014). Development of a prokaryotic universal primer for simultaneous analysis of bacteria and archaea using next-generation sequencing. *PLoS One* 9:e105592. 10.1371/journal.pone.0105592 25144201 PMC4140814

[B102] Tan Y. Cui Y. Li H. Kuang A. Li X. Wei Y. (2017). Diversity and composition of rhizospheric soil and root endogenous bacteria in Panax notoginseng during continuous cropping practices. *J. Basic Microbiol.* 57 337–344. 10.1002/jobm.201600464 28060404

[B103] Thauer R. K. (2010). Functionalization of methane in anaerobic microorganisms. *Angew. Chem. Int. Ed.* 49 6712–6713. 10.1002/anie.201002967 20672272

[B104] Tokida T. Fumoto T. Cheng W. Matsunami T. Adachi M. Katayanagi N. (2010). Effects of free-air CO 2 enrichment (FACE) and soil warming on CH 4 emission from a rice paddy field: impact assessment and stoichiometric evaluation. *Biogeosciences* 7 2639–2653. 10.5194/bg-7-2639-2010

[B105] Umadevi P. Anandaraj M. Srivastav V. Benjamin S. (2018). Trichoderma harzianum MTCC 5179 impacts the population and functional dynamics of microbial community in the rhizosphere of black pepper (*Piper nigrum* L.). *Braz. J. Microbiol.* 49 463–470. 10.1016/j.bjm.2017.05.011 29229530 PMC6066733

[B106] Uroz S. Buée M. Murat C. Frey-Klett P. Martin F. (2010). Pyrosequencing reveals a contrasted bacterial diversity between oak rhizosphere and surrounding soil. *Environ. Microbiol. Rep.* 2 281–288. 10.1111/j.1758-2229.2009.00117.x 23766079

[B107] Vaksmaa A. van Alen T. A. Ettwig K. F. Lupotto E. Valè G. Jetten M. S. (2017). Stratification of diversity and activity of methanogenic and methanotrophic microorganisms in a nitrogen-fertilized Italian paddy soil. *Front. Microbiol.* 8:2127. 10.3389/fmicb.2017.02127 29180985 PMC5693880

[B108] Van Groenigen K. J. Osenberg C. W. Hungate B. A. (2011). Increased soil emissions of potent greenhouse gases under increased atmospheric CO_2_. *Nature* 475 214–216. 10.1038/nature10176 21753852

[B109] van Teeseling M. C. Pol A. Harhangi H. R. van der Zwart S. Jetten M. S. den Camp H. J. O. (2014). Expanding the verrucomicrobial methanotrophic world: description of three novel species of *Methylacidimicrobium* gen. nov. *Appl. Environ. Microbiol.* 80 6782–6791. 10.1128/AEM.01838-14 25172849 PMC4249049

[B110] Verhoeven E. Decock C. Barthel M. Bertora C. Sacco D. Romani M. (2018). Nitrification and coupled nitrification-denitrification at shallow depths are responsible for early season N2O emissions under alternate wetting and drying management in an Italian rice paddy system. *Soil Biol. Biochem.* 120 58–69. 10.1016/j.soilbio.2018.01.032

[B111] Vogel T. M. Simonet P. Jansson J. K. Hirsch P. R. Tiedje J. M. Van Elsas J. D. (2009). TerraGenome: a consortium for the sequencing of a soil metagenome. *Nat. Rev. Microbiol.* 7:252 10.1038/nrmicro2119

[B112] Walters W. A. Jin Z. Youngblut N. Wallace J. G. Sutter J. Zhang W. (2018). Large-scale replicated field study of maize rhizosphere identifies heritable microbes. *Proc. Natl. Acad. Sci. U.S.A.* 115 7368–7373. 10.1073/pnas.1800918115 29941552 PMC6048482

[B113] Wang B. Zhao J. Guo Z. Ma J. Xu H. Jia Z. (2015). Differential contributions of ammonia oxidizers and nitrite oxidizers to nitrification in four paddy soils. *ISME J.* 9 1062–1075. 10.1038/ismej.2014.194 25303715 PMC4409153

[B114] Wang H. Zeng Y. Guo C. Bao Y. Lu G. Reinfelder J. R. (2018). Bacterial, archaeal, and fungal community responses to acid mine drainage-laden pollution in a rice paddy soil ecosystem. *Sci. Tot. Environ.* 616 107–116. 10.1016/j.scitotenv.2017.10.224 29107775

[B115] Wang J. Wang H. Yin T. Xu S. Zhao W. Wang J. (2017). The persistence and performance of phosphate-solubilizing Gluconacetobacter liquefaciens qzr14 in a cucumber soil. *3 Biotech* 7:294. 10.1007/s13205-017-0926-z 28868221 PMC5577371

[B116] Wang J. Xue C. Song Y. Wang L. Huang Q. Shen Q. (2016). Wheat and rice growth stages and fertilization regimes alter soil bacterial community structure, but not diversity. *Front. Microbiol.* 7:1207. 10.3389/fmicb.2016.01207 27536292 PMC4971054

[B117] Wang P. Marsh E. L. Ainsworth E. A. Leakey A. D. Sheflin A. M. Schachtman D. P. (2017). Shifts in microbial communities in soil, rhizosphere and roots of two major crop systems under elevated CO 2 and O 3. *Sci. Rep.* 7:15019. 10.1038/s41598-017-14936-2 29101364 PMC5670137

[B118] Ward N. L. Challacombe J. F. Janssen P. H. Henrissat B. Coutinho P. M. Wu M. (2009). Three genomes from the phylum Acidobacteria provide insight into the lifestyles of these microorganisms in soils. *Appl. Environ. Microbiol.* 75 2046–2056. 10.1128/AEM.02294-08 19201974 PMC2663196

[B119] Xiong W. Li Z. Liu H. Xue C. Zhang R. Wu H. (2015). The effect of long-term continuous cropping of black pepper on soil bacterial communities as determined by 454 pyrosequencing. *PLoS One* 10:e0136946. 10.1371/journal.pone.0136946 26317364 PMC4552827

[B120] Yang Y. Wang N. Guo X. Zhang Y. Ye B. (2017). Comparative analysis of bacterial community structure in the rhizosphere of maize by high-throughput pyrosequencing. *PLoS One* 12:e0178425. 10.1371/journal.pone.0178425 28542542 PMC5444823

[B121] Yuan J. Yuan Y. Zhu Y. Cao L. (2018). Effects of different fertilizers on methane emissions and methanogenic community structures in paddy rhizosphere soil. *Sci. Tot. Environ.* 627 770–781. 10.1016/j.scitotenv.2018.01.233 29426201

[B122] Yulianti E. Rakhmawati A. (2017). “Screening and characterization of phosphate solubilizing bacteria from isolate of thermophilic bacteria,” in *Proceedings of the 4th International Conference on Research, Implementation, and Education of Mathematics and Science (4th icriems): Research and Education for Developing Scientific Attitude in Sciences and Mathematics*, Vol. 1868 (Melville, NY: AIP Publishing), 090015.

[B123] Yvon-Durocher G. Allen A. P. Bastviken D. Conrad R. Gudasz C. St-Pierre A. (2014). Methane fluxes show consistent temperature dependence across microbial to ecosystem scales. *Nature* 507 488–491. 10.1038/nature13164 24670769

[B124] Zhang J. Zhou X. Chen L. Chen Z. Chu J. Li Y. (2016). Comparison of the abundance and community structure of ammonia oxidizing prokaryotes in rice rhizosphere under three different irrigation cultivation modes. *World J. Microbiol. Biotechnol.* 32:85. 10.1007/s11274-016-2042-3 27038955

