# Supplementary material for: 16S rRNA Gene Amplicon Based Metagenomic Signatures of Rhizobiome Community in Rice Field During Various Growth Stages
Source: Front Microbiol. 2019 Sep 20;10:2103. doi: 10.3389/fmicb.2019.02103 (PMC6764247; doi:10.3389/fmicb.2019.02103)
Supplement: Supplementary file 1 [file Data_Sheet_1.pdf]

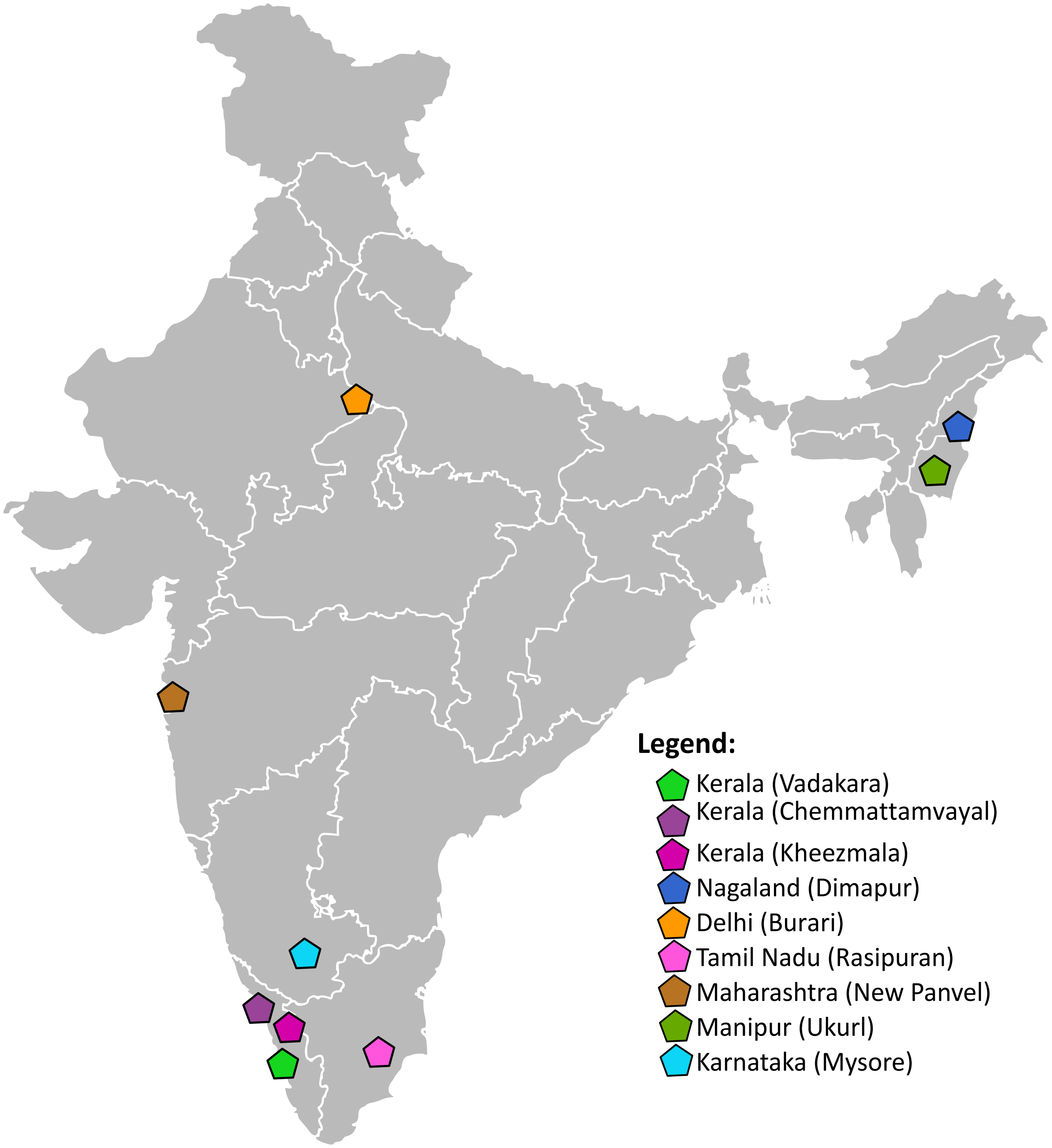

Figure S1: Graphical representation of sampling location within the study.

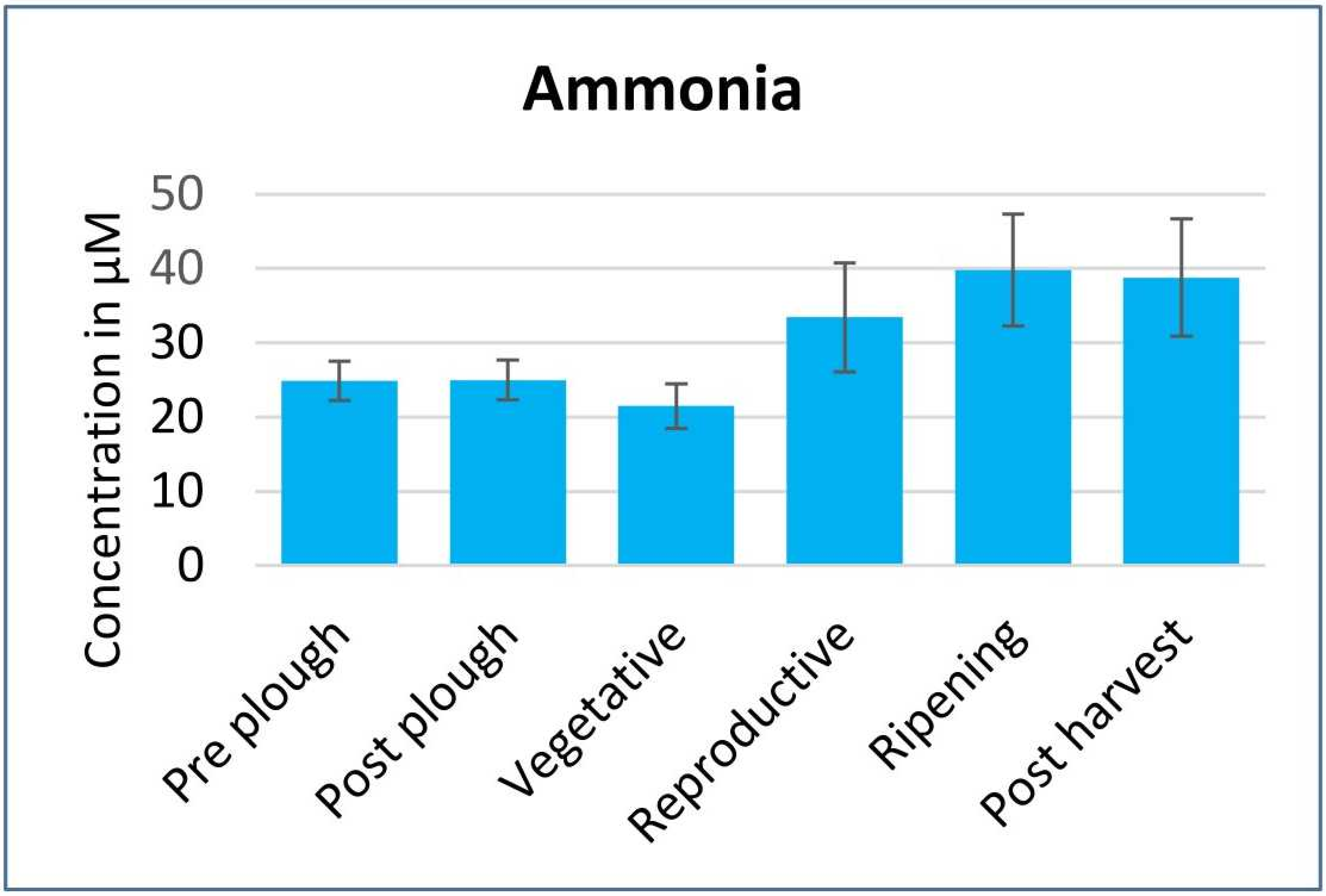

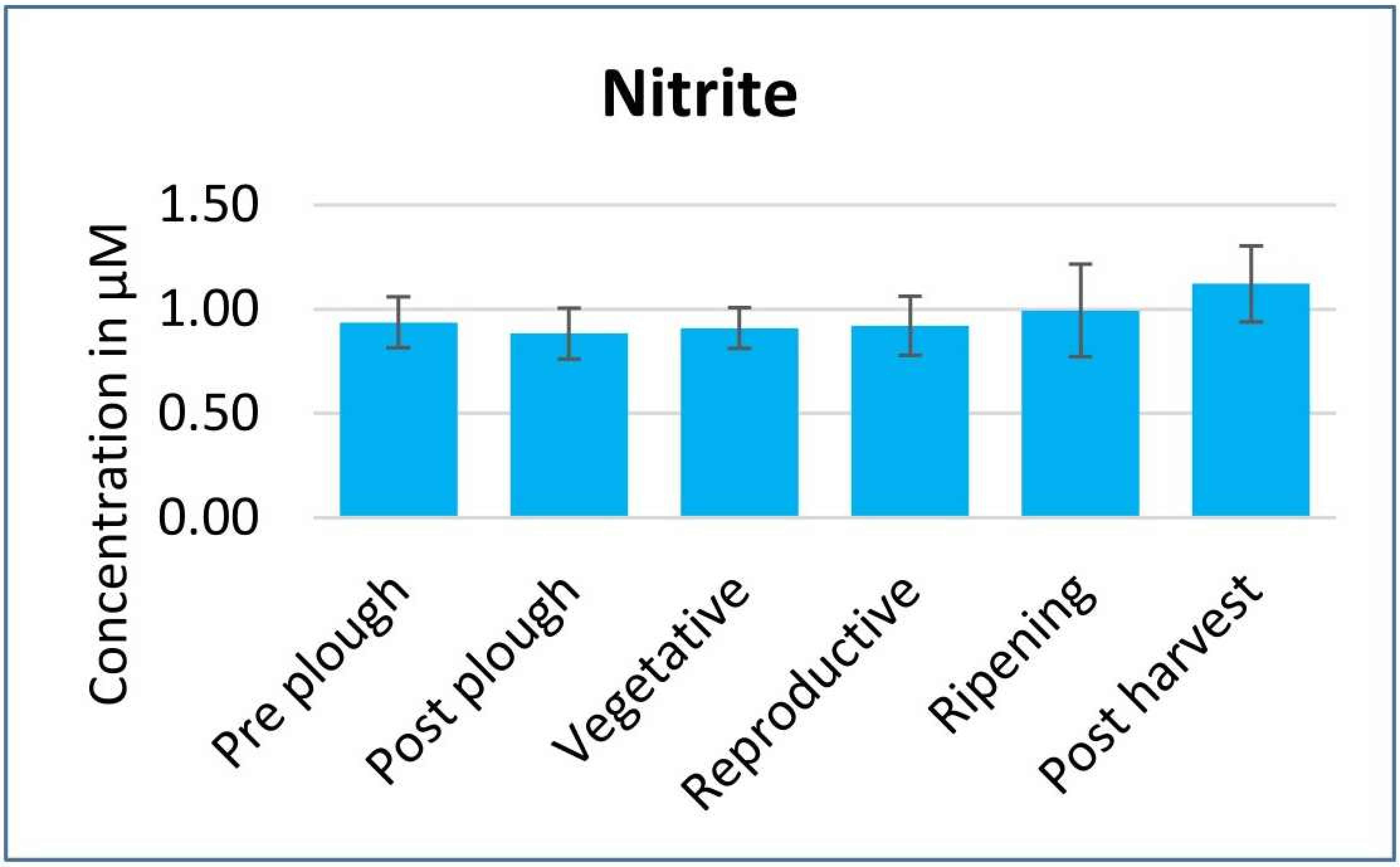

Figure S2: Ammonia and nitrite concentration (µM): The concentration of ammonia had a sharp rise during the cropped stages. Nitrite concentration also rose during cropped stages, however, to a much lesser extent as compared to ammonia.

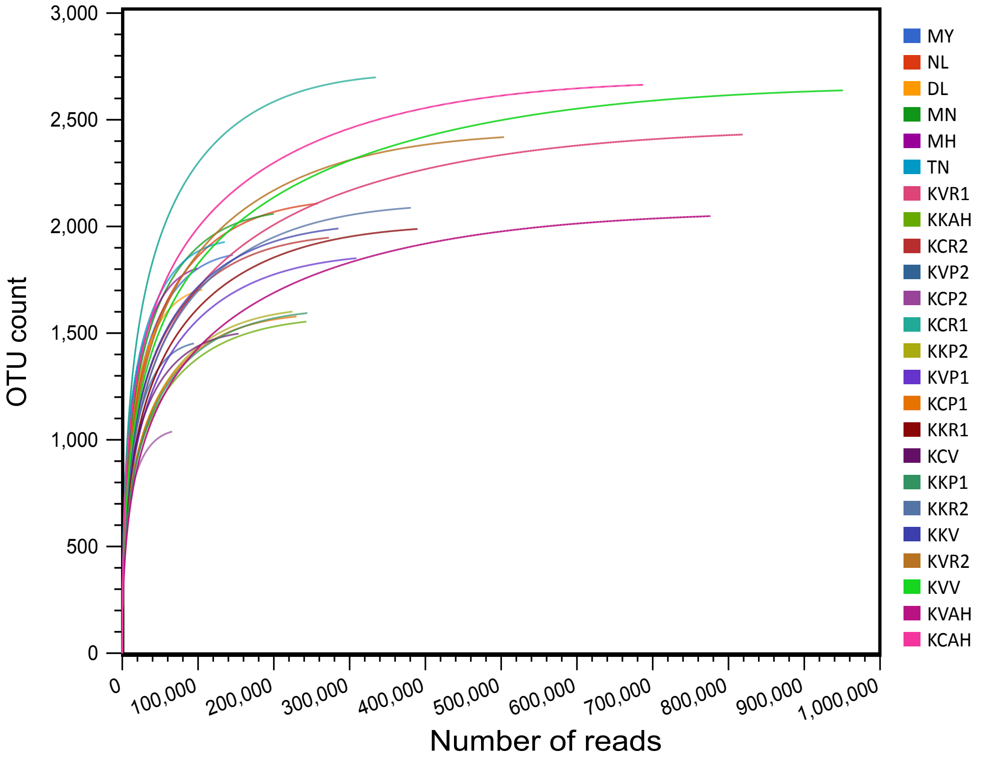

Figure S3: Rarefaction curve of the samples show plateau formation indicating sufficient sequencing depth.

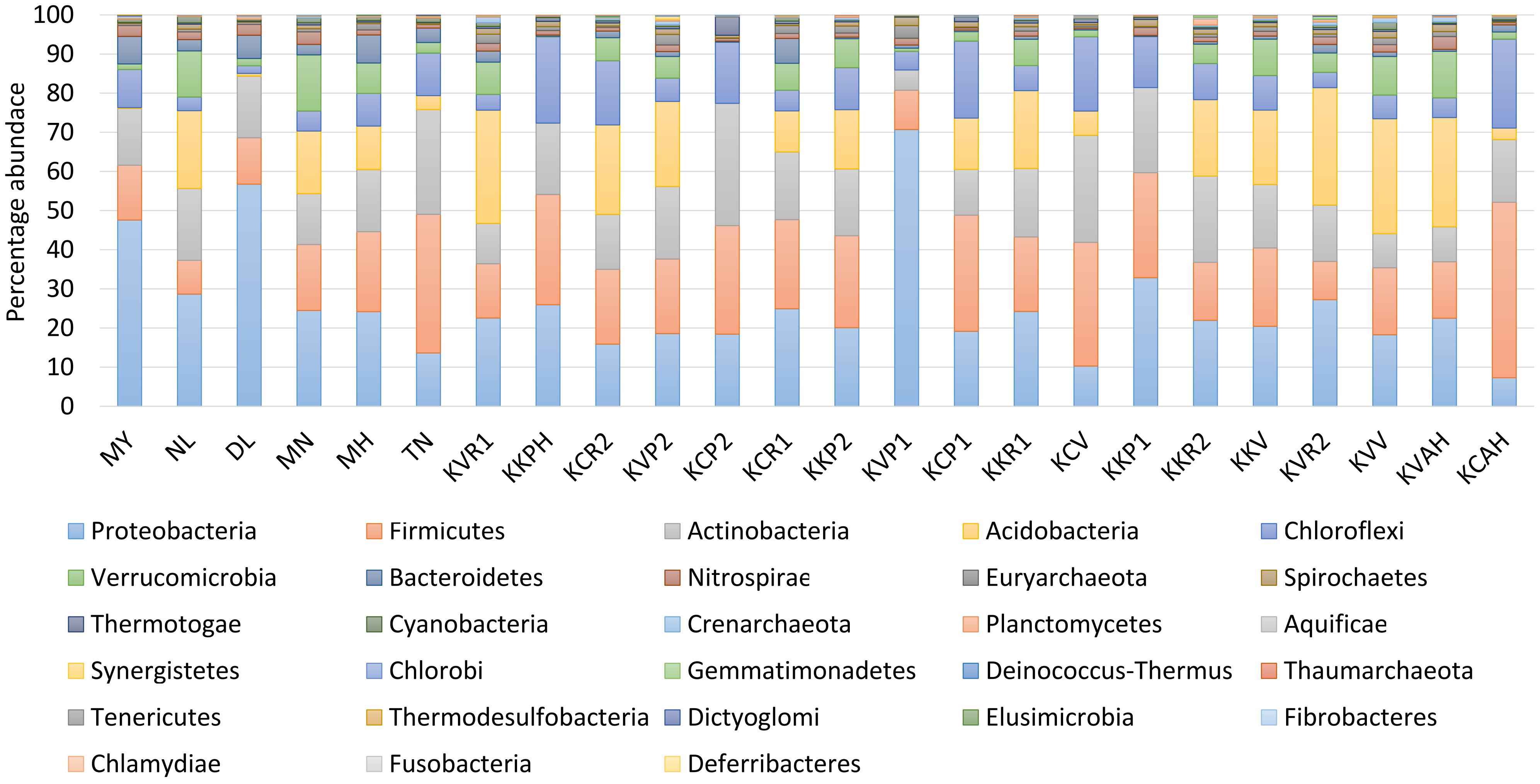

Figure S4: Archeal (Crenarchaeota, Euryarchaeota, and, Thaumarchaeota) and bacterial phyla stack bar chart

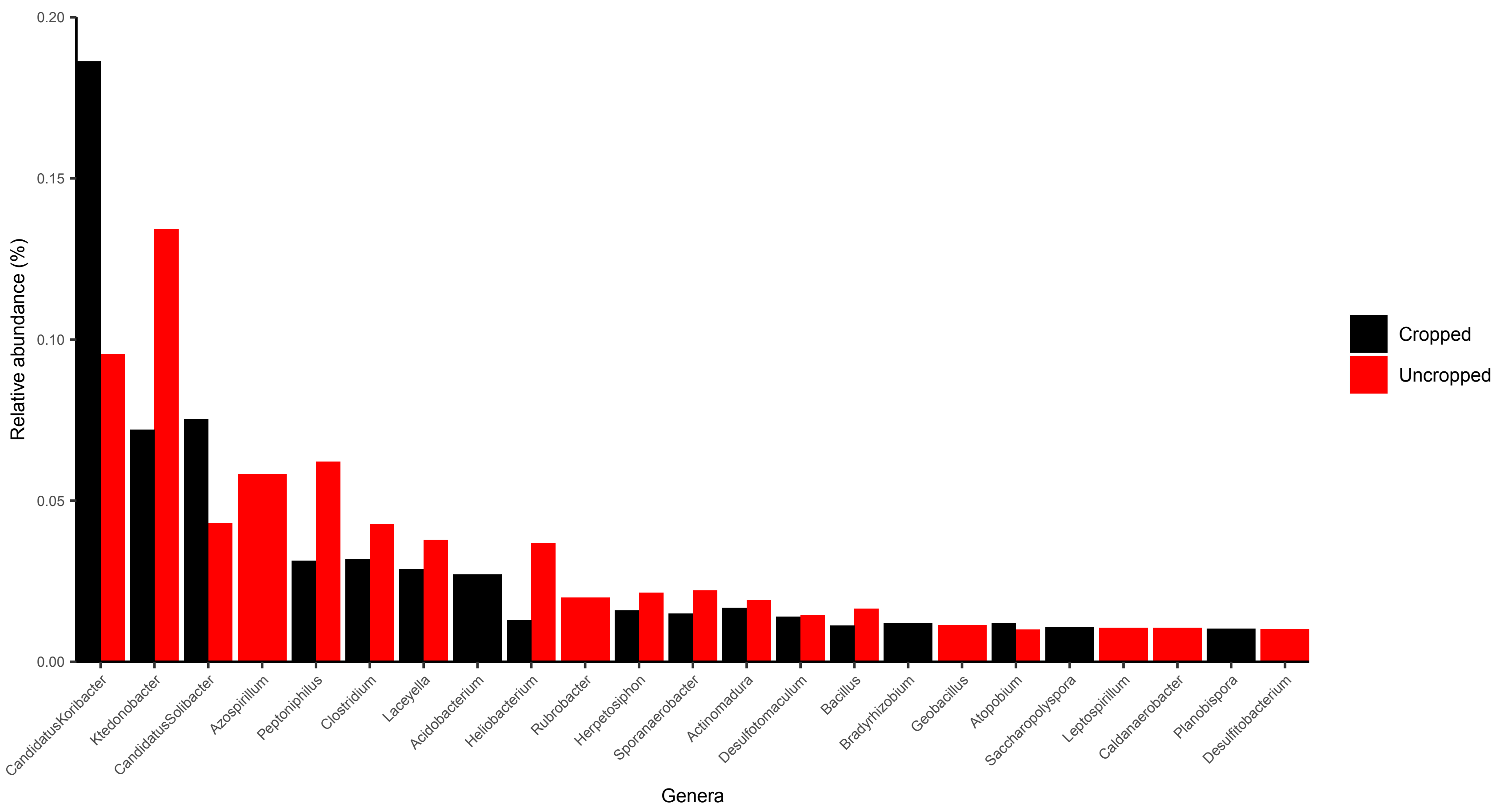

Figure S5: Twenty -three genera were found to have a relative abundance of above 1% in any sample

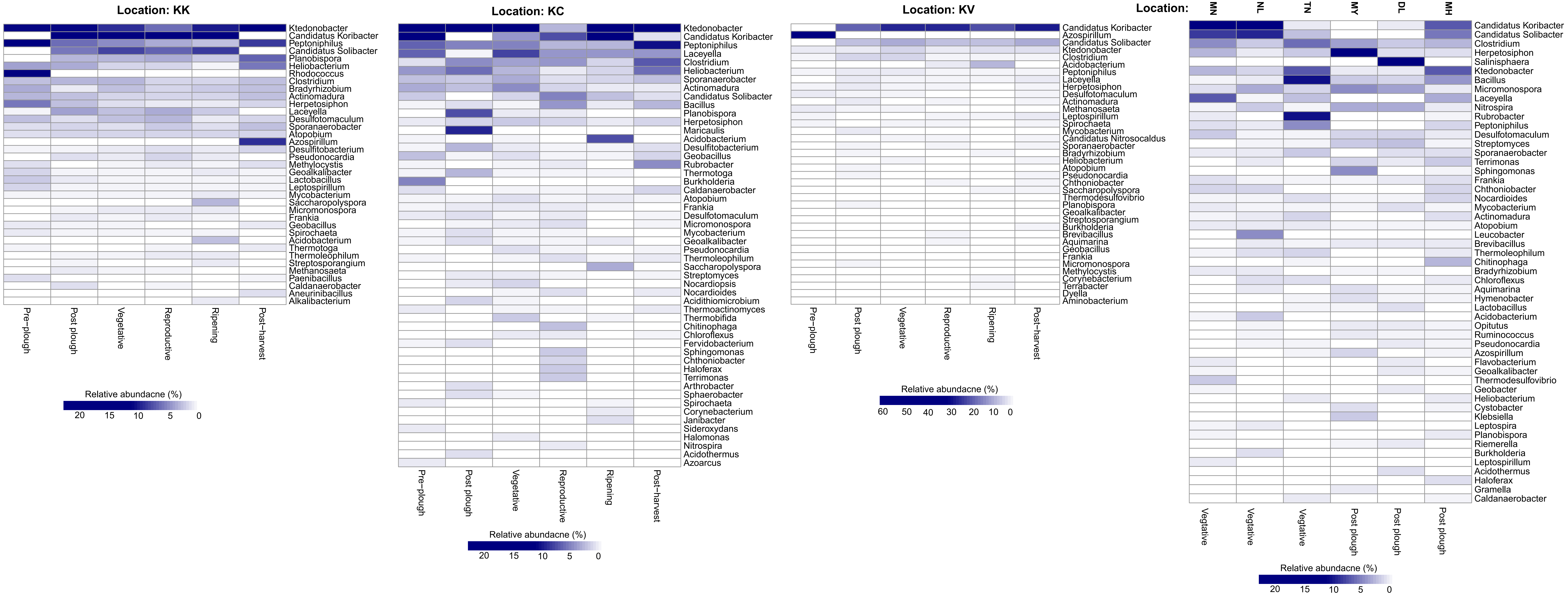

Figure S6: Heatmap representation of dominant (>11% relative abundance) genus at various cultivation stage in different locations.

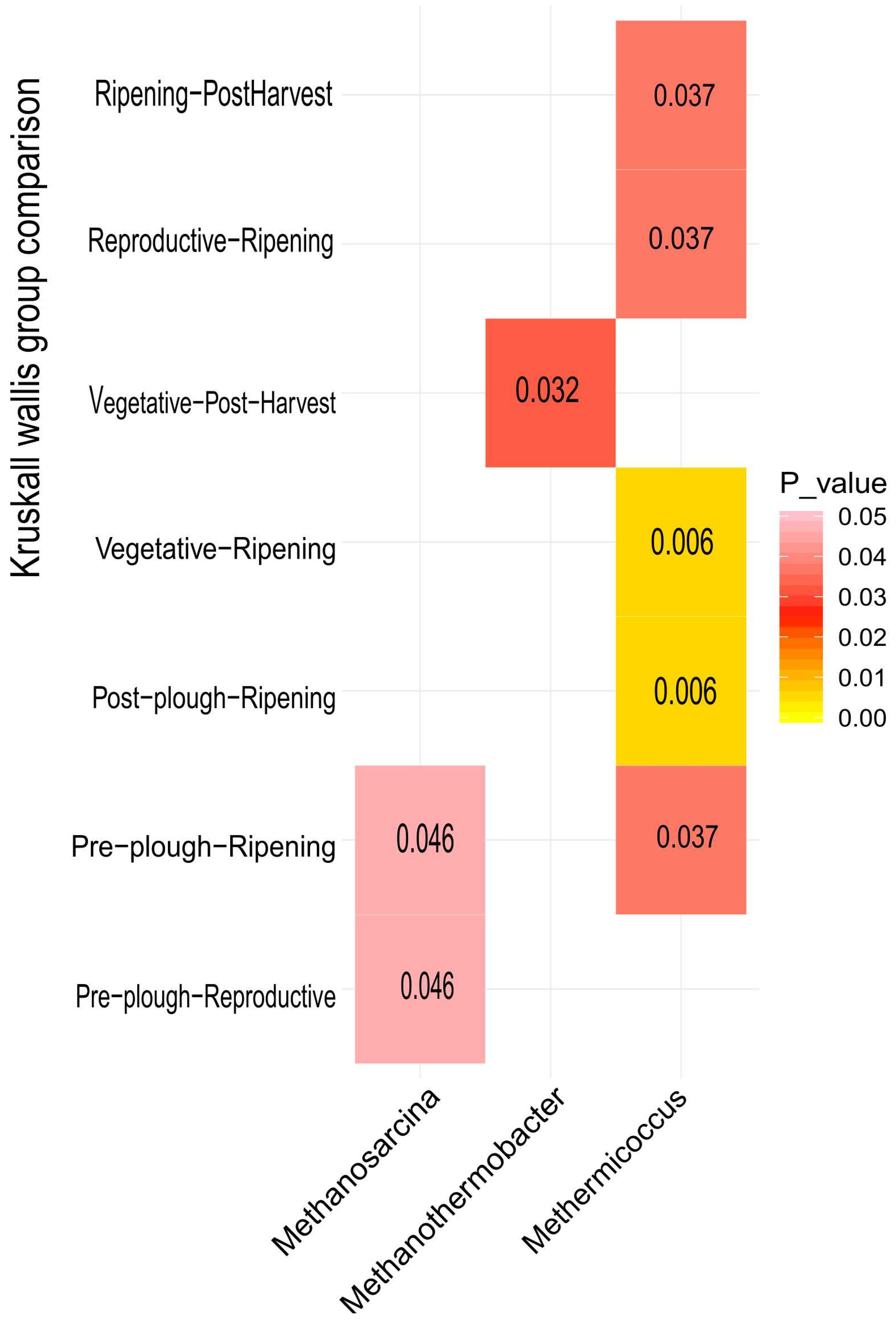

Figure S7: Significant changes of OTU proportion among the groups. Kruskall Wallis comparison. P-value <0.05.

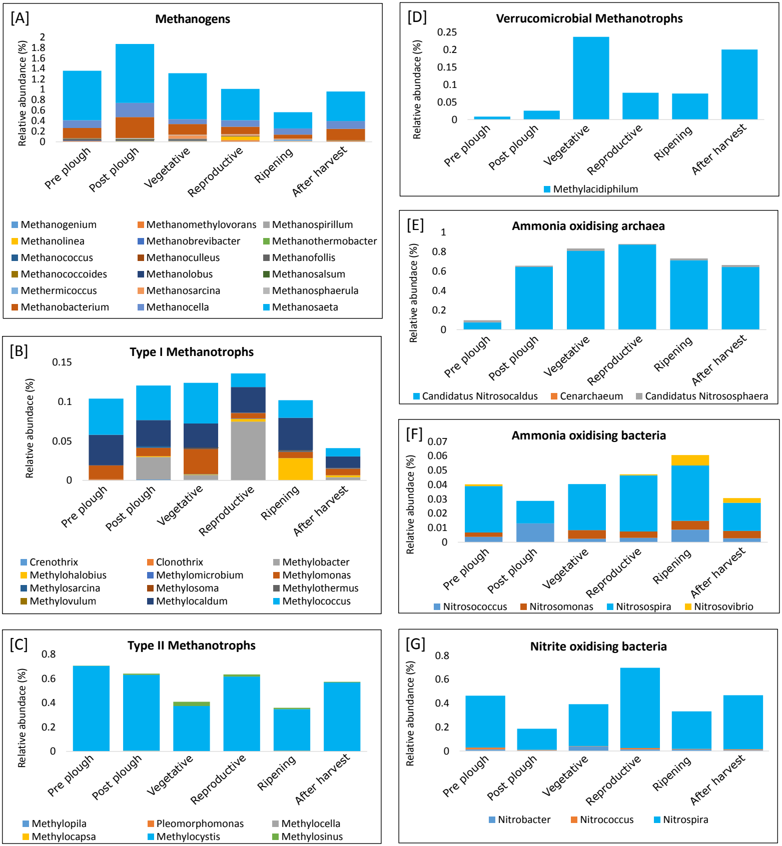

Figure S8: Relative abundance bar chart of [A] methanogens, [B] methanotrophs, [C] ammonia oxidizers and [D] nitrite oxidizers.

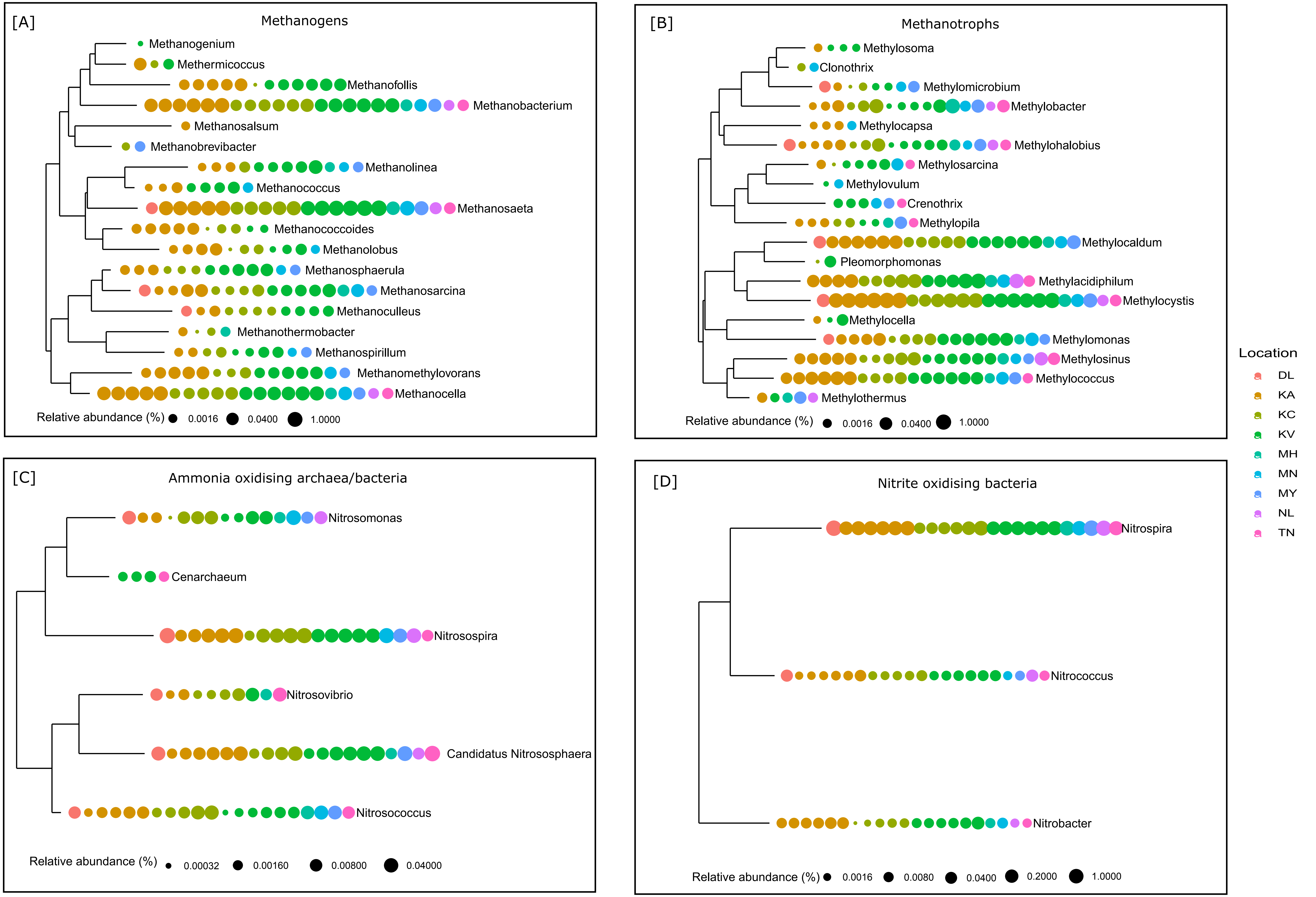

Figure S9: Phylogenetic tree representation of [A] methanogens, [B] methanotrophs, [C] ammonia oxidizers and [D] nitrite oxidizers.

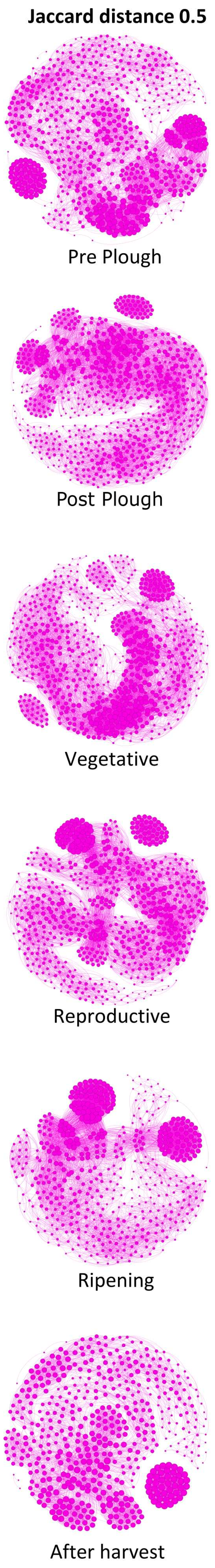

Figure S10: Network complexity map for 0.5 jaccard distance.

Table S1: Summary of descriptive statistics: The raw 16S rRNA gene amplicon samples after quality control had over 2.7 x10^8 bp covering over 0.8 million sequences. The mean GC content had little change after quality control (57.33 ± 0.55).

		Pre Quality Control				Post Quality Control			
Samples	**MG-RAST id**	**bp Count**	**Sequences**	**Mean**	**Mean GC**	**bp Count**	**Sequences**	**Mean**	**Mean GC**
NL	mgm4758101.3	85713814	249777	343	57	7594091	34842	218	57
DL	mgm4758122.3	35772800	102324	350	57	4358290	18533	235	57
MN	mgm4758113.3	68682636	193710	355	57	6881801	29227	235	57
MH	mgm4758109.3	33666969	95650	352	57	4358289	18500	236	57
KVR1	mgm4758110.3	282936617	792550	357	57	15508019	68501	226	57
KKPH	mgm4758107.3	82996827	235076	353	58	6250112	27676	226	58
KCR2	mgm4758115.3	90237018	263850	342	58	7169520	33025	217	57
KVP2	mgm4758116.3	33406435	91513	365	58	4278381	16700	256	57
KCP2	mgm4758111.3	22895936	63718	359	59	2844779	11301	252	58
KCR1	mgm4758106.3	114542703	323871	354	57	9781609	41968	233	57
KKP2	mgm4758114.3	79046941	217389	364	58	6337258	26191	242	57
KVP1	mgm4758117.3	103002592	298914	345	57	9094676	41268	220	57
KCP1	mgm4758118.3	77119738	222450	347	59	6115019	27275	224	58
KKR1	mgm4758121.3	136837318	377230	363	58	8987558	37321	241	57
TN	mgm4758112.3	45085761	131121	344	58	5555129	22831	243	58
KCV	mgm4758108.3	52168250	148732	351	59	5198619	21896	237	58
KKP1	mgm4758105.3	85352169	236272	361	58	6954858	29304	237	57
KKR2	mgm4758119.3	130963548	368574	355	58	8995413	39428	228	57
KKV	mgm4758120.3	99396412	276090	360	58	7792282	32063	243	57
KVR2	mgm4758103.3	175731859	487824	360	57	11446769	48880	234	57
KVV	mgm4758102.3	329568367	920823	358	57	23601732	87006	271	58
KVAH	mgm4758099.3	269158188	751122	358	58	14694503	65122	226	57
KCAH	mgm4758100.3	224806236	665378	338	59	18986392	71005	267	59
MY	mgm4758104.3	49246146	141163	349	57	5040095	21995	229	57
	Total	**2708335280**	**7655121**	**353.45± 7.33**	**57.75± 0.72**	**207825194**	**871858**	**236.5± 13.7**	**57.33± 0.55**

`

Table S2: Tabulated chart of uncultured/unclassified taxa relative abundance in each cultivation stage in different locations.

species	KVP1	KVP2	KVV	KVR1	KVR2	KVPH	KCP1	KCP2	KCV	KCR1	KCR2	KCPH	KKP1	KKP2	KKV	KKR1	KKR2	KKPH	MYS_R	NLVS_R	DLS_R	MNS_R	MHS_R	TNS_R
uncultured Achromobacter sp.	0.000683	0	0.000291	0.002614	0.007563	0	0	0	0	0	0	0.013788	0	0	0.001518	0.011222	0.000689	0	0.002884	0	0	0	0.001406	0
uncultured Acidovorax sp.	0	0	0.01061	0.000327	0.001621	0	0	0	0	0.000958	0	0.000492	0.001295	0.001735	0.000506	6.68E-05	0.000344	0	0.099502	0.00122	0	0.00454	0.002812	0
uncultured actinobacterium	0	0	0	0	0.00054	0	0	0	0	0.027301	0	0	0	0	0	0	0	0	0	0	0	0	0	0
uncultured Afipia sp.	0	0	0.000291	0	0.00081	0	0	0	0	0.000479	0	0	0	0	0	0.000134	0	0	0	0	0	0.000757	0	0
uncultured alpha proteobacterium	0.644804	0.671945	1.077305	0.853144	1.793981	0.841512	0.159443	0.335322	0.318776	1.206054	0.943467	0.364406	0.625656	0.714934	0.738937	0.138143	0.922706	0.539562	5.475521	1.85549	3.87618	0.515339	1.275381	1.714279
uncultured ammonia-oxidizing bacterium	0	0	0	0	0	0	0	0	0	0	0	0	0	0	0	0	0	0	0	0	0	0.001513	0	0.001217
uncultured Antarctic sea ice bacterium	0	0	0	0	0	0	0	0	0	0	0	0	0	0	0	0	0.000344	0	0.005768	0.00061	0	0	0	0
uncultured archaeon	0.081967	0.750576	0.452172	0.18396	0.098045	0.154926	0.074453	0.138074	0.145185	0.06083	0.377347	0.075343	0.349745	0.292105	0.302155	0.019907	0.073679	0.082666	0.044704	0.006712	0.010493	0.151348	0.029529	0.695931
uncultured Azoarcus sp.	0.001366	0	0.000291	0	0.00054	0.002389	0.694664	0	0	0	0	0	0	0.000578	0.000506	0	0	0.004468	0	0.002441	0	0	0	0
uncultured Azospira sp.	0.00888	0	0	0	0	0	0	0	0	0	0	0	0	0	0	0	0	0	0.001442	0	0	0	0	0
uncultured Azospirillum sp.	0	0	0	0	0.00027	0	0	0	0	0	0	0	0	0	0	0	0	0	0	0	0	0	0	0
uncultured Bacillus sp.	0	0	0.000145	0.00049	0	0.001536	0.168574	0.004931	0	0.000479	0.000396	0.000492	0.002591	0.001157	0.043526	0.0002	0.001033	0.001117	0.002884	0	0	0.003027	0	0.001217
uncultured bacterium	24.56541	29.00263	27.73916	28.69044	39.5027	27.54074	18.04581	14.8528	18.38592	34.35578	17.2518	24.95691	29.2452	25.93315	29.94569	5.976298	37.77931	28.95204	48.45771	41.40532	43.13746	32.42172	45.71123	24.97567
uncultured Bartonella sp.	0	0	0	0	0.00027	0	0	0	0	0	0	0	0	0	0	6.68E-05	0	0	0	0	0	0	0	0
uncultured beta proteobacterium	0.502729	0.403882	0.581675	0.454182	0.571254	0.347559	1.085193	0.054243	0.076801	1.309512	0.232153	0.328458	0.167101	0.454064	0.752603	0.162993	1.007058	0.109476	2.173192	1.069607	3.091291	1.428723	1.220541	0.413666
uncultured Bradyrhizobium sp.	0.004098	0.062548	0.008721	0.017971	0.023768	0.003754	0.001405	0.004931	0.004208	0.045502	0.000198	0.003447	0.034975	0.008098	0.013665	0.004876	0.023068	0.012288	0.001442	0.004271	0.004197	0.005297	0.084369	0.006083
uncultured Burkholderia sp.	0.000683	0	0.001017	0.006045	0.025929	0.000682	0.000702	0	0	0.001437	0	0.001477	0.001295	0	0.003543	0.001536	0.001377	0	0.001442	0.048202	0	0.003784	0.012655	0
uncultured Burkholderiales bacterium	0.008197	0.003574	0.01061	0.010293	0.018907	0.003924	0	0	0	0.163809	0.033674	0.003447	0.006477	0.006941	0.036441	0.002472	0.071957	0.012288	0.207657	0.34596	0.167891	0.024972	0.113898	0.046233
uncultured Cellvibrio sp.	0	0	0	0	0.00054	0	0	0	0	0	0	0	0	0	0	0	0	0	0	0	0	0	0	0
uncultured Clostridium sp.	0.017076	0.051826	0.005523	0.001634	0.00135	0.004948	0.007024	0.054243	0.037874	0.022512	0.032684	0.006894	0.018135	0.013882	0.011135	0.000334	0.003787	0.00782	0.096618	0.002441	0.006296	0.017405	0.033748	0.0292
uncultured Comamonadaceae bacterium	0.004098	0.001787	0.004506	0.00147	0.02674	0.001365	0.002107	0	0.003156	0.169556	0.005744	0.010834	0.002591	0.001157	0.014678	0.000868	0.001721	0.001117	0.031725	0.012203	0.075551	0.060539	0.111086	0.0292
uncultured Cupriavidus sp.	0	0	0	0	0	0	0	0	0	0	0	0	0	0	0	6.68E-05	0	0	0	0.00061	0	0.001513	0	0
uncultured cyanobacterium	0.013661	0.001787	0.004796	0.00147	0.002971	0.000682	0.004214	0.009862	0	0.028259	0.027533	0.00197	0.002591	0.000578	0.005567	0.000334	0.004476	0.002234	0.044704	0.056135	0.056663	0.00454	0.012655	0.063266
uncultured Dehalococcoides sp.	0.168032	0.046464	0.020784	0.022056	0.009183	0.029177	0.0295	0.078899	0.049447	0.009579	0.006141	0.027577	0.046633	0.017353	0.015184	0.001536	0.005853	0.006703	0.002884	0.00061	0	0.015135	0.01828	0.002433
uncultured delta proteobacterium	5.700781	4.759011	3.37232	3.444924	4.512233	6.167504	4.354117	2.874895	2.510231	2.849411	1.906941	4.209386	9.164626	4.750033	4.499927	0.93761	4.932002	4.83707	1.721826	3.430308	3.278069	6.557898	2.45655	2.283677
uncultured Desulfobacteraceae bacterium	0	0	0.000291	0.001307	0.041325	0.002389	0.004214	0	0.001052	0.001916	0.000396	0.025114	0	0	0.001012	6.68E-05	0.003443	0	0	0.00061	0.002099	0.001513	0	0
uncultured Desulfobacterium sp.	0.002049	0.003574	0.001017	0.001797	0.001621	0.001365	0.004917	0.014794	0.003156	0.002874	0.009706	0.004924	0.002591	0.002892	0.003543	0.0002	0.001033	0.004468	0.00721	0.00122	0.039874	0.005297	0	0.00365
uncultured Desulfobacula sp.	0	0	0	0	0	0	0	0	0	0	0	0	0	0	0	0	0	0	0.001442	0.00061	0.002099	0	0	0
uncultured Desulfobulbaceae bacterium	0.000683	0	0.001163	0.000163	0	0.000853	0.002107	0.004931	0.006312	0.008622	0.008319	0.004924	0.009067	0.024294	0.013159	0.000134	0	0.014522	0.080756	0.00061	0.010493	0.012108	0.011249	0
uncultured Desulfobulbus sp.	0.000683	0	0.034011	0.00098	0.022688	0.001194	0	0	0.001052	0.006706	0.000198	0.006402	0	0	0.000506	0	0.004476	0	0	0	0	0.03027	0.002812	0
uncultured Desulfovibrio sp.	0.000683	0	0.000291	0	0.00054	0.000512	0.00281	0	0.001052	0.008143	0	0.000985	0.002591	0.001157	0.001518	0.000134	0	0	0	0.002441	0	0.000757	0	0.00365
uncultured Desulfovibrionales bacterium	0.013661	0.019658	0.021947	0.004738	0.008643	0.015527	0.007726	0.014794	0.012625	0.019638	0.011687	0.033486	0.014249	0.008676	0.012147	0.004142	0.055087	0.008937	0.015863	0.002441	0.023085	0.012108	0.016874	0.013383
uncultured Enterobacter sp.	0	0	0	0.00098	0.001621	0.000171	0	0	0	0.000958	0.012281	0	0	0	0	0	0.000344	0	0.002884	0	0	0	0	0.0292
uncultured Enterobacteriaceae bacterium	0	0	0.000436	0.000163	0.00135	0	0	0	0	0	0	0	0	0	0	0.000534	0	0	0	0	0	0.005297	0	0
uncultured epsilon proteobacterium	0.099726	0.296656	0.075435	0.115016	0.095884	0.070297	0.536626	0.147936	0.046291	0.067056	0.060811	0.060078	0.031088	0.03297	0.021763	0.00648	0.038905	0.030162	0.099502	0.032949	0.054565	0.097619	0.097025	0.096116
uncultured euryarchaeote	0	0	0	0	0.001621	0	0	0	0	0	0	0.00197	0	0	0	0	0.035462	0	0	0	0	0	0	0
uncultured Firmicutes bacterium	0.005464	0	0.001163	0.003267	0.002701	0.001706	0.001405	0.004931	0	0.001916	0.000198	0	0.006477	0.001735	0.003543	0.000735	0.005164	0.004468	0.018747	0.003051	0.010493	0.00227	0.001406	0.001217
uncultured forest soil bacterium	0.239069	1.095484	11.55501	19.61071	0.959383	15.42857	1.002311	1.267321	0.781686	1.383274	0.184217	0.740139	1.30183	21.9501	8.648605	83.53196	1.24221	0.340717	0.149975	0.11715	0.266527	1.866875	0.240452	0.18615
uncultured gamma proteobacterium	1.135238	1.229516	1.040242	0.987275	1.560888	0.899012	0.742426	2.618472	1.509716	1.877096	0.443705	1.334023	1.082916	0.470839	0.586089	0.218037	0.672061	1.314834	1.821328	1.702341	1.775446	1.397696	1.366781	0.833414
uncultured Geobacter sp.	0.096994	0.032168	0.03372	0.116323	0.039164	0.088724	0.036524	0.004931	0.016833	0.047897	0.065763	0.090609	0.051814	0.006941	0.146775	0.006613	0.025822	0.005586	0.059125	0.053694	0.270724	0.091565	0.033748	0.121666
uncultured Geobacteraceae bacterium	0	0	0	0	0.010534	0	0	0	0	0	0	0.001477	0	0	0	0	0.000344	0	0	0	0	0	0	0
uncultured Helicobacter sp.	0	0	0.000145	0	0	0	0	0	0	0	0	0	0	0	0	0	0.000344	0	0	0	0	0	0	0
uncultured Hyphomicrobium sp.	0.007514	0.028593	0.005523	0.002287	0.004322	0.002047	0.000702	0	0	0.005269	0.004358	0.000492	0.029793	0.013304	0.007592	0.001269	0.002066	0.014522	0.015863	0.007932	0.002099	0.001513	0.035154	0.01095
uncultured Klebsiella sp.	0	0	0.000291	0.010293	0.00081	0.000171	0.000702	0	0	0.033049	0.026147	0.002955	0	0	0	0	0.000344	0	0.48886	0	0	0.022702	0.004218	0.019467
uncultured Legionella sp.	0	0	0.000291	0	0	0	0.000702	0	0	0	0	0	0	0	0	0	0	0	0	0	0	0	0	0
uncultured Magnetospirillum sp.	0	0	0.004796	0.001634	0.002431	0.000171	0.002107	0	0	0.000958	0.000198	0.000492	0	0.001735	0	0.000267	0	0	0	0	0	0	0	0.001217
uncultured marine bacterium	0.188523	0.037529	0.038517	0.121224	0.071035	0.03003	0.052679	0.03945	0.02525	0.204043	0.068933	0.019698	0.151556	0.052637	0.033404	0.006613	0.040627	0.168683	0.282645	0.129353	0.119622	0.083998	0.077338	0.328499
uncultured marine microorganism	0	0	0	0.002451	0	0	0	0	0.001052	0.000479	0.001387	0.001477	0	0	0	0.000134	0.000344	0	0	0.00122	0	0	0	0
uncultured Methanomicrobiaceae archaeon	0.001366	0	0.000291	0	0.00027	0	0	0	0.00526	0	0	0	0	0	0	0	0	0.001117	0	0	0	0	0	0
uncultured Methanosarcina sp.	0	0.001787	0.05465	0.004411	0	0	0	0	0	0.000958	0	0	0.001295	0	0.013159	0	0	0	0	0	0	0	0.004218	0
uncultured Methanosarcinales archaeon	0.004781	0	0.006105	0.00049	0.00027	0.000512	0	0	0.001052	0	0.00099	0	0.007772	0.001735	0.000506	0.000267	0	0	0	0	0	0.003027	0	0
uncultured Methylobacter sp.	0.000683	0	0.000145	0.00049	0	0.000341	0	0	0	0.101063	0.007923	0.000492	0	0	0.008098	0.000134	0	0	0	0.00061	0	0.00227	0.177175	0.023117
uncultured Methylobacterium sp.	0.000683	0	0.000145	0.001144	0.00054	0	0.000702	0	0	0.002874	0.001585	0.000985	0	0.000578	0.003543	6.68E-05	0.000689	0.002234	0.002884	0.00061	0.002099	0	0.002812	0.01095
uncultured Methylocystaceae bacterium	0.008197	0.001787	0.000872	0.005065	0.002971	0.000512	0.003512	0.009862	0.016833	0.007185	0.001387	0.00197	0.037565	0.004049	0.028849	0.00147	0.025133	0.006703	0	0.00061	0	0.000757	0.004218	0
uncultured microorganism	0	0	0	0	0	0	0	0	0	0	0	0	0	0	0	0	0	0	0	0.00061	0	0	0	0
uncultured Nitrosomonadaceae bacterium	0.124999	0.003574	0.04215	0.037086	0.043756	0.001877	0.008429	0	0.00526	0.04215	0.001783	0.04038	0.031088	0.012147	0.037453	0.013293	0.08022	0.026811	0.001442	0.049423	0.004197	0.043891	0.023905	0.002433
uncultured Nitrosomonadales bacterium	0.043716	0.010723	0.022674	0.016337	0.036193	0.018086	0.03512	0.128211	0.084165	0.096753	0.004952	0.095041	0.018135	0.145185	0.009616	0.003607	0.00482	0.048036	0.111039	0.177556	0.293809	0.014378	0.032342	0.015817
uncultured Nitrosomonas sp.	0	0	0.000145	0	0	0.005289	0	0	0	0	0	0	0	0	0	0	0	0	0	0	0	0	0	0
uncultured Ochrobactrum sp.	0.025273	0	0.002035	0.000653	0.002431	0.000853	0.000702	0	0	0.000958	0.00099	0.001477	0.005181	0.000578	0.002531	0.000134	0.001033	0.00782	0	0.00183	0.006296	0	0.001406	0.002433
uncultured organism	0.59904	0.521829	0.559873	0.911796	0.591241	0.566297	0.09693	0.143005	0.088374	0.261519	0.134894	0.210272	0.255185	0.237733	0.293044	0.090247	0.475469	0.803199	0.873891	0.384399	0.237146	0.20659	0.250295	0.225083
uncultured prokaryote	0	0	0.000145	0	0	0	0	0	0	0	0.000792	0	0	0	0	0	0	0	0.001442	0	0	0	0	0
uncultured proteobacterium	0.108606	0.109012	0.286768	0.891864	0.567203	0.282551	0.249349	0.024656	0.143081	0.521123	0.128159	0.441227	0.314771	0.380026	0.334546	0.070474	0.361508	0.045801	0.161511	0.588802	0.543547	0.550906	0.426064	0.082733
uncultured Pseudomonas sp.	0.001366	0	0.00407	0.001307	0.007293	0.000853	0.001405	0.009862	0	0.000958	0.000396	0.005909	0	0.004049	0.006073	0	0.012395	0	0.005768	0.010983	0.008395	0	0	0.002433
uncultured Ralstonia sp.	0.00888	0.01251	0.017732	0.010946	0.021068	0.000682	0.033715	0.019725	0.016833	0.021554	0.010102	0.037918	0.011658	0.006363	0.025812	0.011623	0.012739	0.010054	0.005768	0.032949	0.033578	0.006054	0.029529	0.001217
uncultured Rhizobiales bacterium	0.433057	0.01251	0.01061	0.006208	0.011344	0.001536	0.00281	0	0	0.148482	0.000792	0.006402	3.187865	0.004627	0.020751	0.011556	0.013427	0.021225	0.010094	0.018915	0.006296	0.008324	0.074526	0.00365
uncultured Rhizobium sp.	0	0	0	0	0	0.000171	0	0	0	0.000479	0.002971	0	0	0	0	6.68E-05	0.000344	0	0	0	0	0	0.002812	0
uncultured Rhodobacteraceae bacterium	0.001366	0.003574	0.00407	0.000653	0.002431	0.014332	0.004917	0	0	0.008143	0.00416	0	0.001295	0	0.004555	0.0002	0.001721	0	0.001442	0	0.023085	0.016648	0.001406	0.01095
uncultured Rhodobacterales bacterium	0.003415	0.003574	0.006977	0.00098	0.00054	0.001024	0	0	0	0.000479	0.000792	0	0.002591	0.000578	0	0.000134	0.002066	0.002234	0.005768	0.00183	0	0	0.004218	0.001217
uncultured Rhodocyclaceae bacterium	0.00888	0.073271	0.087644	0.147854	0.313312	0.204236	0.037929	0	0	0.119743	0.007725	0.028069	0.024612	0.107009	0.220163	0.015498	0.138062	0	0.030283	0.439924	0.365163	0.133186	0.178581	0.024333
uncultured Rhodospirillaceae bacterium	0.020492	0.234108	0.303337	0.229052	0.387318	0.121142	0.266206	0.004931	0.12204	0.104895	0.053878	0.105875	0.081607	0.111636	0.178661	0.035404	0.144947	0.065909	0.122576	0.029288	0.018888	0.074917	0.067495	0.042583
uncultured Rickettsia sp.	0	0.001787	0	0	0.00027	0	0	0	0	0	0	0	0	0	0	0	0	0.002234	0	0	0	0	0	0
uncultured Roseobacter sp.	0	0	0	0	0	0.000171	0	0	0	0	0	0	0	0	0	0	0.000344	0	0	0	0	0	0	0
uncultured rumen bacterium	0.000683	0	0.001889	0.001307	0.001621	0.000682	0	0	0.004208	0.004311	0.00099	0.00197	0	0	0	6.68E-05	0.001033	0	0	0.002441	0	0	0	0
uncultured Serratia sp.	0	0	0	0.004248	0.004862	0	0	0	0	0	0.000198	0.002462	0.007772	0	0	0.000601	0.023756	0	0	0	0	0	0	0
uncultured Sinorhizobium sp.	0	0	0	0	0.00027	0	0	0	0	0.000958	0.000198	0	0	0	0	0	0	0	0	0	0	0	0.059058	0
uncultured soil bacterium	0.381145	1.876441	0.48066	0.359098	0.781389	0.465971	0.089204	0.231767	0.426087	0.938787	0.380319	0.428424	0.29275	0.247566	0.384652	0.148096	0.456877	0.217836	1.02675	0.901813	1.441763	0.279237	0.496372	1.105947
uncultured Streptococcus sp.	0	0.001787	0.000145	0.000163	0	0	0	0	0	0.000479	0	0	0	0	0	0	0.000344	0	0	0	0	0	0	0
uncultured Streptomyces sp.	0	0.001787	0.00436	0.01307	0.003511	0.003754	0.003512	0	0.037874	0.002874	0.010696	0	0	0.003471	0.002024	0.000468	0.010329	0	0	0.006102	0	0.006054	0	0.009733
uncultured SUP05 cluster bacterium	0.001366	0	0.008139	0.000163	0	0	0.000702	0	0	0	0.000198	0.000492	0	0.000578	0.001012	0	0.000344	0.001117	0	0	0	0.001513	0	0.001217
uncultured Thiobacillus sp.	0.133879	0.003574	0.000872	0.002941	0.002161	0.006825	0.005619	0	0	0	0	0.003447	0	0	0	0	0.000344	0	0	0	0	0.011351	0	0
uncultured Verminephrobacter sp.	0	0	0.000727	0.000327	0	0	0	0	0	0.000958	0	0	0	0	0	0	0	0	0	0.003661	0	0.040107	0	0
uncultured Verrucomicrobia bacterium	0	0	0.021366	0.001307	0.00027	0	0	0	0.003156	0.003353	0.011885	0	0	0.001735	0.007592	0.000601	0.001377	0	0	0.013423	0	0.003784	0.001406	0.012167
unidentified	0.129097	0.105438	0.068313	0.069271	0.046997	0.052381	0.008429	0.014794	0.011573	0.148482	0.068735	0.006894	0.093265	0.031813	0.032898	0.00648	0.032019	0.089368	0.902733	0.260537	0.283316	0.049188	0.147646	0.220216
unidentified cyanobacterium CLg1	0.000683	0	0	0	0.00027	0	0	0	0.001052	0	0	0.000492	0	0	0	0	0	0	0	0	0	0	0	0
unidentified marine eubacterium	0.001366	0.005361	0.006395	0.001307	0.00108	0.001024	0	0	0.002104	0.017722	0.000594	0.002955	0.001295	0.002314	0.00658	0.000534	0.002066	0	0.017305	0.022576	0.050367	0.021189	0.019686	0.002433
unidentified microorganism	0	0	0	0	0	0	0	0	0	0	0.000198	0	0	0	0	0	0	0	0	0	0	0	0	0.001217
unidentified proteobacterium	0	0	0.009593	0.00049	0.002161	0	0.000702	0	0	0.019159	0.001387	0.007879	0	0.014461	0.001012	0.000534	0.001721	0	0	0.009152	0	0.00227	0.007031	0
unidentified sulfate-reducing bacterium	0.002049	0.005361	0.011192	0.014214	0.009994	0.017915	0.015453	0.024656	0.032614	0.017722	0.004754	0.034963	0.005181	0.005784	0.013665	0.001336	0.008952	0.001117	0.001442	0.013423	0.012592	0.047675	0.014062	0.027983

`

Table S3: Comparative analysis of the difference between cropped and uncropped stages having significance ($P < 0.05$) difference yield 143 genera.

**Sr. No**	**Genus**	**Cropped (% abundance)**	**Uncropped (% abundance)**	**Kruskal *P* value**
1	*Candidatus Solibacter*	6.777	3.181	0.037
2	*Acidobacterium*	2.745	0.185	0.003
3	*Heliobacterium*	1.554	3.831	0.047
4	*Herpetosiphon*	1.473	2.587	0.024
5	*Saccharopolyspora*	1.206	0.302	0.038
6	*Chthoniobacter*	0.844	0.314	0.019
7	*Nocardioides*	0.64	0.316	0.024
8	*Thermobifida*	0.527	0.130	0.031
9	*Brevibacillus*	0.500	0.110	0.047
10	*Aquimarina*	0.488	0.040	0.009
11	*Chitinophaga*	0.404	0.050	0.012
12	*Terrimonas*	0.304	0.032	0.047
13	*Geodermatophilus*	0.289	0.094	0.009
14	*Halomonas*	0.267	0.065	0.031
15	*Dyella*	0.230	0.046	0.031
16	*Isosphaera*	0.214	0.097	0.037
17	*Actinocorallia*	0.153	0.283	0.031
18	*Acinetobacter*	0.137	0.020	0.042
19	*Hymenobacter*	0.137	0.069	0.038
20	*Pseudomonas*	0.135	0.031	0.038
21	*Selenomonas*	0.135	0.317	0.009
22	*Methylacidiphilum*	0.130	0.078	0.046
23	*Pantoea*	0.101	0.007	0.015
24	*Sphingobacterium*	0.095	0.013	0.012
25	*Ralstonia*	0.09	0.043	0.047
26	*Rothia*	0.072	0.040	0.009
27	*Flavobacterium*	0.070	0.025	0.031
28	*Kineococcus*	0.070	0.002	0.003
29	*Chromobacterium*	0.069	0.003	0.029
30	*Pedobacter*	0.068	0.006	0.009
31	*Cytophaga*	0.067	0.022	0.009
32	*Ectothiorhodospira*	0.055	0	0.01
33	*Sulfurihydrogenibium*	0.055	0	0.031
34	*Novosphingobium*	0.052	0.001	0.001
35	*Collinsella*	0.051	0.134	0.009
36	*Thiomonas*	0.049	0.009	0.038
37	*Cyanothece*	0.048	0.002	0.011
38	*Dehalococcoides*	0.048	0.129	0.024
39	*Castellaniella*	0.046	0	0.031
40	*Microbispora*	0.045	0.019	0.03

`

41	*Bdellovibrio*	0.041	0.009	0.005
42	*Acetivibrio*	0.04	0.092	0.047
43	*Desulfonatronovibrio*	0.038	0.004	0.02
44	*Prosthecochloris*	0.036	0.061	0.015
45	*Desulfomonile*	0.035	0.008	0.037
46	*Methanosarcina*	0.035	0.003	0.006
47	*Microcoleus*	0.034	0.001	0.002
48	*Natronorubrum*	0.034	0.007	0.046
49	*Tetrasphaera*	0.034	0.018	0.024
50	*Pelomonas*	0.033	0.008	0.014
51	*Achromobacter*	0.031	0	0.002
52	*Variovorax*	0.031	0.002	0.011
53	*Flexibacter*	0.03	0.003	0.024
54	*Petrotoga*	0.027	0.1	0.019
55	*Carboxydothermus*	0.025	0.009	0.03
56	*Pimelobacter*	0.024	0.004	0.03
57	*Bordetella*	0.023	0	0.002
58	*Cupriavidus*	0.023	0.004	0.013
59	*Deinococcus*	0.023	0.004	0.009
60	*Desulfobulbus*	0.022	0.003	0.009
61	*Methylosinus*	0.022	0.006	0.03
62	*Parabacteroides*	0.021	0.003	0.015
63	*Eggerthella*	0.02	0.005	0.037
64	*Kytococcus*	0.02	0	0.033
65	*Desulfatibacillum*	0.018	0.005	0.007
66	*Gluconacetobacter*	0.018	0.007	0.009
67	*Herbaspirillum*	0.017	0.004	0.012
68	*Gardnerella*	0.016	0.003	0.03
69	*Nonomuraea*	0.016	0.006	0.047
70	*Streptomonospora*	0.016	0	0.042
71	*Leptothrix*	0.015	0.001	0.03
72	*Lysobacter*	0.015	0.001	0.014
73	*Melittangium*	0.014	0.005	0.015
74	*Desulfohalobium*	0.013	0.002	0.039
75	*Elusimicrobium*	0.013	0.002	0.019
76	*Halostagnicola*	0.012	0.001	0.004
77	*Spirulina*	0.012	0.004	0.023
78	*Desulfonatronum*	0.011	0.003	0.008
79	*Sinorhizobium*	0.01	0	0.042
80	*Acidaminococcus*	0.009	0.035	0.024
81	*Acidovorax*	0.009	0.001	0.048
82	*Pseudanabaena*	0.009	0.001	0.015
83	*Acaryochloris*	0.008	0	0.005
84	*Chlorogloeopsis*	0.008	0.001	0.013

`

85	*Legionella*	0.008	0.006	0.038
86	*Phormidium*	0.008	0.001	0.03
87	*Pyramidobacter*	0.008	0.002	0.038
88	*Cronobacter*	0.007	0.001	0.007
89	*Dolichospermum*	0.007	0.001	0.014
90	*Limnothrix*	0.007	0	0.02
91	*Sarcina*	0.007	0.003	0.03
92	*Viridibacillus*	0.007	0	0.012
93	*Acidiphilium*	0.006	0	0.001
94	*Asticcacaulis*	0.006	0	0.033
95	*Flectobacillus*	0.006	0	0.004
96	*Swaminathania*	0.006	0	0.005
97	*Thermincola*	0.006	0.003	0.048
98	*Escherichia*	0.005	0.001	0.018
99	*Microtetraspora*	0.005	0.002	0.046
100	*Saccharothrix*	0.005	0	0.033
101	*Segniliparus*	0.005	0.004	0.042
102	*Xylella*	0.005	0	0
103	*Dermacoccus*	0.004	0	0.021
104	*Kozakia*	0.004	0	0.026
105	*Mitsuaria*	0.004	0	0.031
106	*Wautersiella*	0.004	0	0.026
107	*Actinokineospora*	0.003	0	0.003
108	*Afipia*	0.003	0	0.005
109	*Capnocytophaga*	0.003	0.001	0.042
110	*Coxiella*	0.003	0	0.003
111	*Kluyvera*	0.003	0	0.029
112	*Rhodovibrio*	0.003	0	0.01
113	*Sphaerotilus*	0.003	0	0.029
114	*Spirobacillus*	0.003	0	0.01
115	*Aeriscardovia*	0.002	0	0.021
116	*Allochromatium*	0.002	0	0.012
117	*Ancylobacter*	0.002	0	0.013
118	*Caedibacter*	0.002	0	0.027
119	*Cellvibrio*	0.002	0.013	0.034
120	*Duganella*	0.002	0	0.04
121	*Granulicatella*	0.002	0.001	0.021
122	*Labrenzia*	0.002	0	0.029
123	*Leucobacter*	0.002	0	0.001
124	*Roseateles*	0.002	0	0.013
125	*Scytonema*	0.002	0	0.04
126	*Sinobacter*	0.002	0	0.012
127	*Spirosoma*	0.002	0	0.005
128	*Aquaspirillum*	0.001	0	0.031

`

129	*Bergeyella*	0.001	0	0.029
130	*Candidatus Midichloria*	0.001	0	0.013
131	*Desulfurivibrio*	0.001	0	0.029
132	*Methylopila*	0.001	0	0.039
133	*Oligella*	0.001	0	0.029
134	*Pseudacidovorax*	0.001	0	0.005
135	*Rhodobium*	0.001	0	0.005
136	*Rhodothalassium*	0.001	0	0.04
137	*Acholeplasma*	0	0	0.029
138	*Ensifer*	0	0	0.029
139	*Leuconostoc*	0	0.001	0.031
140	*Methylophilus*	0	0	0.029
141	*Neisseria*	0	0	0.029
142	*Photorhabdus*	0	0	0.04
143	*Xenophilus*	0	0	0.029
